# Heterologous Expression of Either Human or Soya Bean Ferritins in Budding Yeast Reveals Common Functions Protecting Against Oxidative Agents and Counteracting Double-Strand Break Accumulation

**DOI:** 10.3390/biom15030447

**Published:** 2025-03-20

**Authors:** Nuria Pujol Carrión, Maria Ángeles de la Torre-Ruiz

**Affiliations:** IRBLleida-Department Basic Medical Science, University of Lleida, Alcalde Rovira Roure No. 80, 25198 Lleida, Spain; nuria.pujol@udl.cat

**Keywords:** human ferritin, soya bean ferritin, iron, oxidative stress, DSB (double-strand break), *Saccharomyces cerevisiae*

## Abstract

Ferritins are globular proteins that, upon self-assembly in nanocages, are capable of bio-safely storing huge concentrations of bioavailable iron. They are present in most cell types and organisms; one of the exceptions is yeast. Heterologous expression of either human or vegetal ferritins in *Saccharomyces cerevisiae* revealed new and unknown functions for soya bean ferritins; validated this model by confirming previously characterized functions in human ferritins and also demonstrated that, like human H chain, vegetal H1, and H2 chains also shown a tendency to localize in the nucleus when expressed in an eukaryotic cell model lacking plastids and chloroplasts. Furthermore, when expressed in the system budding yeast, the four ferritins (human H and L and soya bean H1 and H2 chains) present equivalent and relevant functions as protectors against oxidative damage and against the accumulation of double-strand breaks in the DNA. We present evidence demonstrating that these effects are exclusively observed with oxidative agents that operate through the Fenton reaction, such as H_2_O_2_. Here, we also discuss the ferritin requirement for N-glycosylation to exert these functions. We believe that our approach might contribute to extending the knowledge around ferritin function and its consequent relevance to human health.

## 1. Introduction

Iron is an essential metal for all cells involved in multiple processes, such as cellular respiration, lipid biosynthesis, translation and amino acid biogenesis, DNA replication and repair, oxygen transport, photosynthesis, or nitrogen fixation [1]. Excess or deficiency of iron is harmful to the cells; in fact, dysregulation of iron metabolism results in an elevated number of important diseases [2,3,4]. Excess of free iron generates deleterious reactive oxygen species (ROS) due to their role in Fenton and Haber–Weiss reactions [5]. ROS, such as the hydroxyl radical, H_2_O_2,_ and the superoxide radical, can damage any cell component, and the induction of several types of DNA damage is especially relevant [6,7].

Iron bioavailability is limited since the oxidized Fe^3+^ forms ferric hydroxides with a tendency to create precipitates at a physiological pH of 7.4. In humans, iron deficiency anaemia (IDA) has been reported to be the most frequent nutritional disorder worldwide [8].

Ferritin proteins are the principal iron storage structures in cells. They are expressed in most eubacteria, archaea, plants, animals, and humans, with the exception of yeasts [9].

The main function of ferritins is to store bioavailable iron and, at the same time, to avoid the oxidative effects that free iron provokes in all cells. Ferritin proteins have antioxidant capacity since they protect cells from the Fenton reaction and from the accumulation of hydroxyl radicals [10].

There are two different human ferritins: H (Heavy) and L (Light). The H chain has ferrous oxidase activity and oxidizes Fe^2+^ to Fe^3+,^ whereas the L ferritin is involved in iron nucleation. The H chain contains a dinuclear ferroxidase site located in the four-helix bundle of the subunit, and it catalyses the oxidation of ferrous iron by O_2_-producing hydrogen peroxide. The L-subunit lacks this catalysing centre and contains additional glutamate residues on the internal surface of the protein shell, which produce a microenvironment that facilitates mineralization and the turnover of Fe^3+^ at the H-subunit ferroxidase centre. In general, ferritins form spherical nanocages composed of about 24 subunits in whose interior iron is mineralized and stored [11]. Plant ferritins are similar to animal ferritins [12]. Ferric iron is also in a shell of ferritin subunits as an iron core [13]. Plant ferritins exhibit distinctive characteristics. They are located in plastids and are only constituted by H-type subunits that, as in the case of animal ferritins, also perform nucleation and oxidation of iron atoms [14,15]. Animal and vegetal ferritins can be used and biotechnologically engineered as iron sources with relevant interest in nutrition [16,17].

Other important properties, such as iron donors and chaperones involved in [Fe–S] assembly or their potential use in immunotherapy, have also been attributed to ferritins [18,19]. Some reports have associated human nuclear ferritins with protection against DNA damage caused by iron [20,21] or H_2_O_2_ [22]. Other authors [23] reported that iron oxide nanoparticles provoked persistent DNA damage attributed to reactive oxygen species (ROS) in human melanoma cells, more specifically, double-strand breaks evidenced by phosphorylated H2AX foci. In *S. cerevisiae*, Rad52 foci represent sites of DNA damage repair [24]. Rad52 plays a central role in homologous recombination-based DNA double-strand break repair: DSB, reviewed in [25].

Since the beginning of this century, *Saccharomyces cerevisiae* has been widely engineered with human and plant genes to test their function. We have made use of this strategy to express heterologous proteins in budding yeast and to illustrate the functional characteristics of both human and soya bean ferritins. We show interesting differences between human and soya bean ferritins in cellular localization and protein stability. However, the four H, L, H1, and H2 ferritins display some conserved and similar functions as reducers and counteractors of the negative effects that ROS, dependent on iron, provoke in cell viability and in DNA damage, more specifically in DSB.

## 2. Materials and Methods

### 2.1. Yeast Strains and Plasmids

*Saccharomyces cerevisiae* wild-type strain CML128 background (MATa *leu2-3,112 ura3-52 trp1 his4 can1r*) has been previously described in [26]. Wild-type strains BY4741 (MATa *his3Δ1*, *leu2Δ0*, *met17Δ0*, *ura3Δ0*) and W303-1A (MATa *ade2-1*, *trip1-1*, *leu2-3,2-111*, *his3-11,75*, *ura3*) have been previously described in [27].

Both human and soya bean ferritins cloning into plasmids were previously described in [28]. Human L and H ferritin chains were cloned into the BamHI and SalI sites of the pUG35 vector under the MET25 promoter and fused in frame to the GFP epitope in the C terminus. Both soybean H1 and H2 ferritin chains were cloned into the BamHI and SalI sites of the pUG35 vector. L human and H2 soybean ferritin chains were cloned into PmeI and NotI sites of the integrative vector pMM351, respectively, under the *TetO_7_* promoter and fused in frame to the HA epitope in the C terminus. The centromeric plasmid containing the fusion protein Rad52YFP under its own promoter was described in [29]. Strain *rad52* (MATa, *rad52*::*hph*) is isogenic to BY4741 from EUROSCARF collection, whereas *rad53* strain (MATa *rad53*::*HIS3*, *sml1-1*) is isogenic to W303 [30].

### 2.2. Media and Growth Conditions

Yeasts were grown at 30 °C in SD medium (2% glucose, 0.67% yeast nitrogen base that lacked the corresponding amino acids for plasmid maintenance) plus amino acids [31]. For iron depletion conditions (SD-Fe), SD medium was used with a yeast nitrogen base that is free of iron plus the addition of 80 µM of 4,7-diphenyl-1,10-phenanthrolinedisulfonic acid (BPS) (Sigma, 146617, St. Louis, MO, USA) [32]. Cell cultures were exponentially grown (optical density at 600 nm [OD_600_] of 0.6) or at longer times as indicated at 30 °C.

We present a list of reagents detailing the stock concentration and from which company they were purchased: Cycloheximide (CH) 150 mg/mL (Sigma, C4859); DAPI 2 mg/mL (Sigma, D9541); (N-(3-triethylammoniumpropyl)-4-(p-diethylaminophenylhexatrienyl)) pyridinium dibromide (FM4-64) 3 mg/mL (Invitrogen, Waltham, MA, USA, T-3166); MG132 1 mg/mL (StressMarq Biosciences, Victoria, BC, Canada); Nocodazole 10 mg/mL (Sigma, 497828); Aphidicolin 1 mg/mL (ClinicSciences, Nanterre, France, sc-201535); Tunicamycin 1 mg/mL (Sigma T7765); H_2_O_2_ 30% (*w*/*w*) (Sigma, H1009) and Diamide 1 mg/mL (Sigma D3648). Iron was added as ammonium iron (III) sulphate hexacahydrate [NH_4_Fe(SO_4_)2⋅6H_2_O] (+Fe; F1543; Sigma).

The final concentrations and the incubation times were indicated in each experiment.

### 2.3. Vacuole and Staining

Cells were stained with FM4-64 (N-(3-triethylammoniumpropyl)-4-(p-diethylaminophenylhexatrienyl) pyridinium dibromide as described by our group [33].

### 2.4. Cell Survival

To assay cell viability, cells were grown to mid-log phase at an OD_600_ of 0.6 in SD medium supplemented with the required amino acids. Viability was registered through serial dilutions and plated in triplicate onto YPD plates (2% glucose, 2% peptone, and 1% yeast extract).

### 2.5. Protein Extraction and Immunoblot Analyses

Total yeast protein extracts were prepared as previously described in [34]. The antibodies for western blotting were anti-GFP (no.632381; Living Colours, Danvers, MA, USA) at a dilution of 1:2000 and anti-phospho-glycerate kinase (anti-PGK1) (no.459250; Invitrogen) at a dilution of 1:10.000. The corresponding secondary antibodies were anti-mouse IgG horseradish peroxidase (from sheep) (no.NA931; GE Healthcare, Chicago, IL, USA) for anti-GFP and anti-PGK1. All these antibodies were used as indicated by the manufacturers. The protein–antibody complexes were visualized by enhanced chemiluminescence using the Supersignal substrate (Pierce) in a Chemidoc (Roche Applied Science, Penzberg, Upper Bavaria, Germany).

### 2.6. Fluorescence Microscopy

Cells were visualized under the fluorescence microscope (Olympus BX-51, Wilow Grove, PA, USA) using 60× magnification. Cellular localizations were registered under specific conditions described in the text.

### 2.7. Statistical Analysis

We followed the same procedure as described in [29]. Error bars in the histograms represent the standard deviation (SD) calculated from three independent experiments. The significance of the data was determined by *p*-values from a Student unpaired *t*-test denoted as follows: * 0.05 > *p* > 0.01; ** 0.01 > *p* > 0.001; *** 0.001 > *p* > 0.0001.

## 3. Results

### 3.1. Expression of Human (H and L) and Soya Bean Ferritins (H1 and H2) in Budding Yeast Reveals New Information Concerning In Vivo Localization and Protein Stability

**Figure 1:**
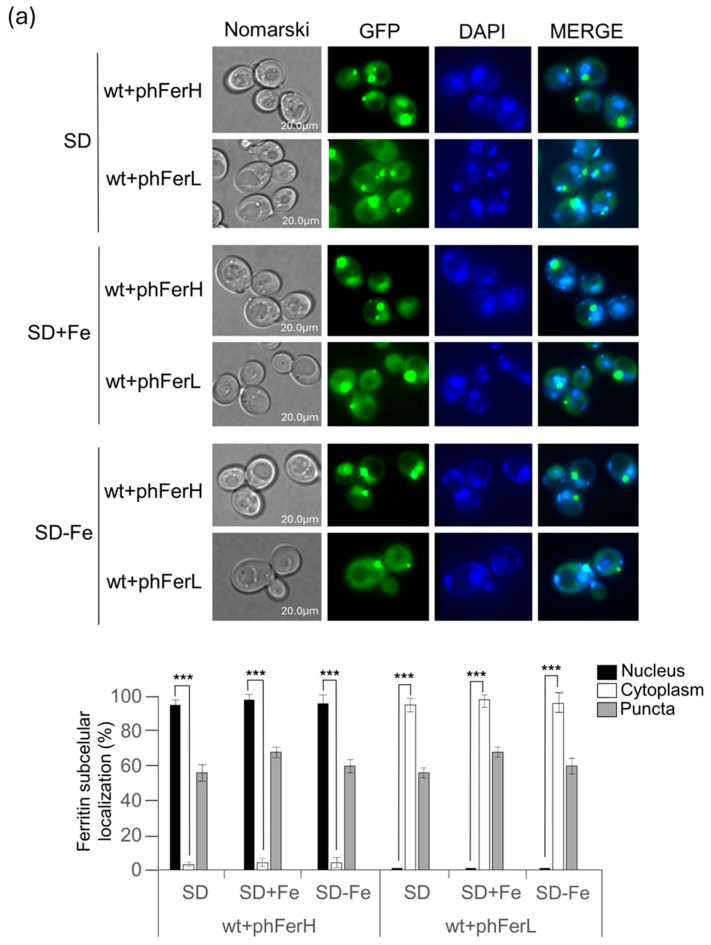

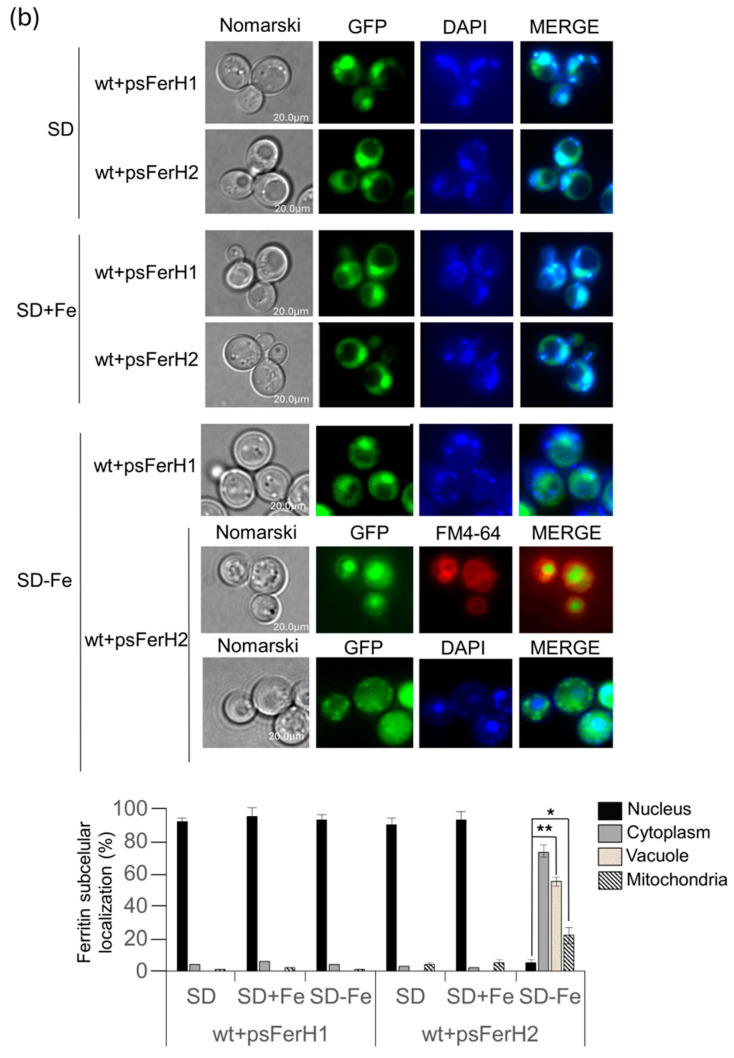
Iron-dependent cellular localization of both human and soya bean ferritins expressed in *S. cerevisiae*. (**a**) Wild-type strains expressing either L or H human ferritins were grown to exponential phase (OD_600_ = 0.6) at 30 °C in either SD medium, SD+8 mM iron or SD minus iron. In vivo observation in the fluorescence microscope of L or H overexpressed proteins fused to GFP. DAPI staining was performed to identify nuclear localization. Ferritin cellular localization was recorded upon each treatment and plotted in histograms. (**b**) As in (**a**), however, the ferritins expressed were either H1 or H2 soya bean chains. For microscopy images in this paper, we have selected representative samples. Error bars in the histograms represent the standard deviation calculated from three independent experiments. The significance of the data was determined by *p*-values from a Student unpaired *t*-test denoted as follows: * = 0.05 > *p* > 0.01; ** = 0.01 > *p* > 0.001; *** = 0.001 > *p* > 0.0001.

In order to ascertain the localization and expression of either human or soya bean ferritins in the eukaryotic model system *Saccharomyces cerevisiae*, we used the previously published plasmids overexpressing each of these proteins that were previously described in [28]. Cultures overexpressing either of both human H or L chains or, alternatively, H1 or H2 soya bean ferritins with or without iron were grown to exponential phase in SD minimum media plus amino acids at 30 °C (normal conditions). Both H and L human ferritin chains are localized in cytoplasmic big puncta. H chain also showed nuclear localization, whereas the L chain only localized in the cytoplasm (Figure 1a). Addition or depletion of iron to the culture media did not provoke any change in these results (Figure 1a). Soya bean ferritins, in turn, did not localize in cytoplasmic puncta in any of the conditions tested. Thus, in normal conditions and in the presence of iron, both soya bean ferritins are mainly localized in the nuclei. When iron was depleted, no change was observed in H1 chain localization. However, part of the H2 ferritin chain translocated both to the vacuole and also to a few dots, which colocalized with mitochondria. (Figure 1b).

Wt cultures transformed with the empty pUG35 plasmid showed a GFP(Green fluorescent protein) diffuse and homogeneous localization all over the cells, and this pattern did not change in any of the treatments conducted in this paper (our unpublished results).

**Figure 2:**
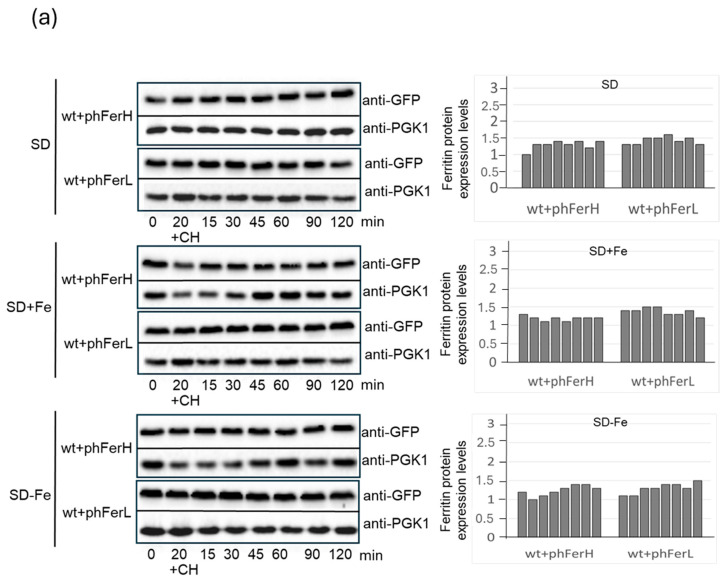

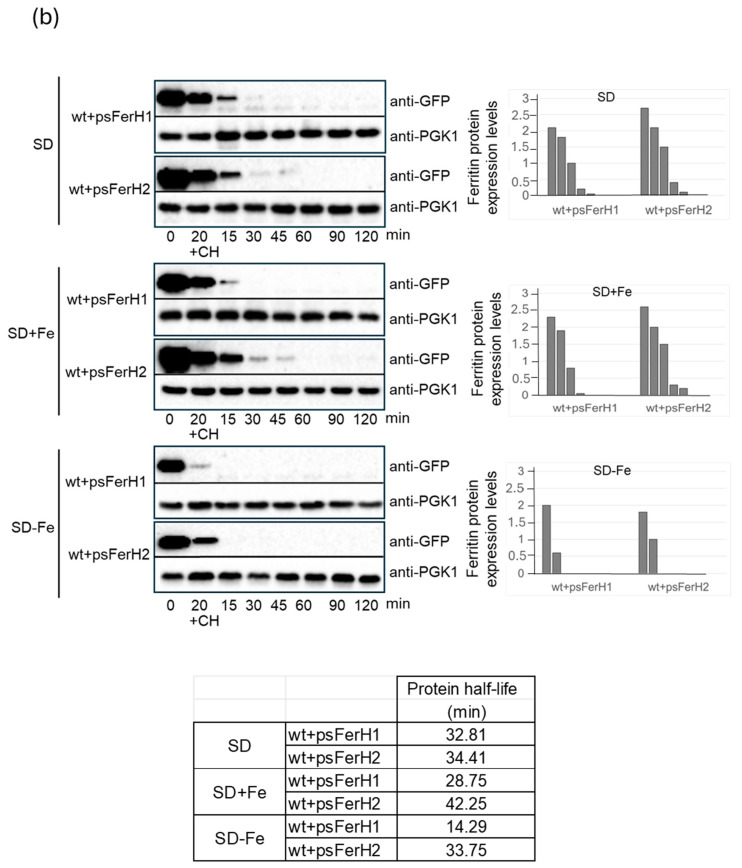
Determination of ferritin stability under several conditions. (**a**) Wild-type cultures expressing each of H or L ferritins were exponentially grown in SD, SD+8 mM iron, or SD-Fe to OD_600_: 0.6. After that, all cultures were treated with 100 µg/mL cycloheximide (+CH), 20 min of treatment was considered as time 0 for calculations. Upon the indicated periods, samples were taken for western blot. (**b**) As in (**a**), however, wt was expressing either H1 or H2 soya bean ferritins. For all the western blot figures, ferritin proteins were detected using anti-GFP antibodies, whereas the loading control, PGK1, was detected using anti-PGK1 antibodies. Histograms represent levels of each ferritin, calculated as the ratio between the values determined with the anti-GFP antibody and those determined with anti-PGK1 as the loading control. Original images of Figure 2 can be found in Supplementary Materials.

Stability assays were conducted in cells grown in SD media plus amino acids to log phase (OD_600_:0.6), then washed and transferred to SD-Fe media, to which cycloheximide was added to block protein synthesis. Our results revealed that human ferritins remained very stable for long periods of time, independently of iron concentrations (Figure 2a). Soya bean ferritins were less stable than human ones upon expression in *S. cerevisiae* (Figure 2b). Thus, in the presence of iron (standard concentrations, SD medium), half-lives of H1 and H2 ferritins were similar. However, iron addition induced a mild increase in the stability of the H2 chain (Figure 2b). Iron starvation provoked a remarkable shortening in soya bean ferritin’s half-life, as compared with standard conditions. Both in conditions of excess and starvation of iron, H2 ferritin presented more stability than the H1 chain (Figure 2b).

Ferritins have been described to depend not only on Fe levels but also on oxidative stress. Therefore, we analyzed human H and L and soya bean H1 and H2 stability upon the addition of H_2_O_2_ as an oxidant agent in the presence or absence of iron in the culture media. We did not observe any effect caused by the addition of H_2_O_2_ in either human or soya bean ferritins (Figure S1). 

### 3.2. In Conditions of Iron Scarcity, Both H1 and H2 Soya Bean Ferritins Are Degraded in Proteasome to Liberate Iron, and Only H2 Ferritin Is Also Degraded Through a Mechanism Dependent on Bulk Autophagy

**Figure 3:**
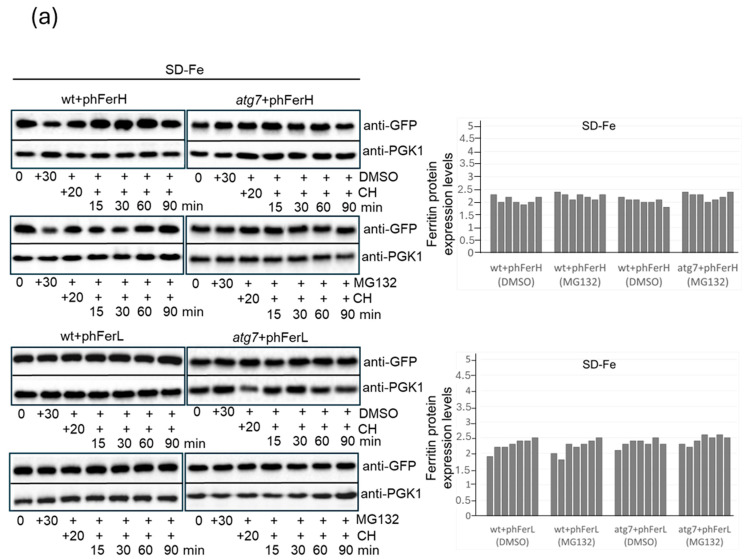

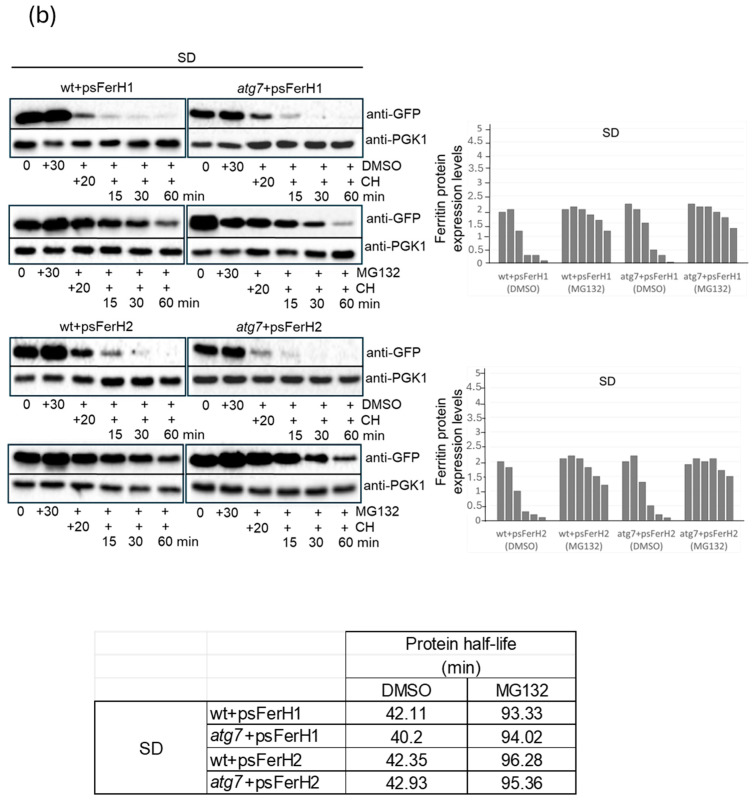

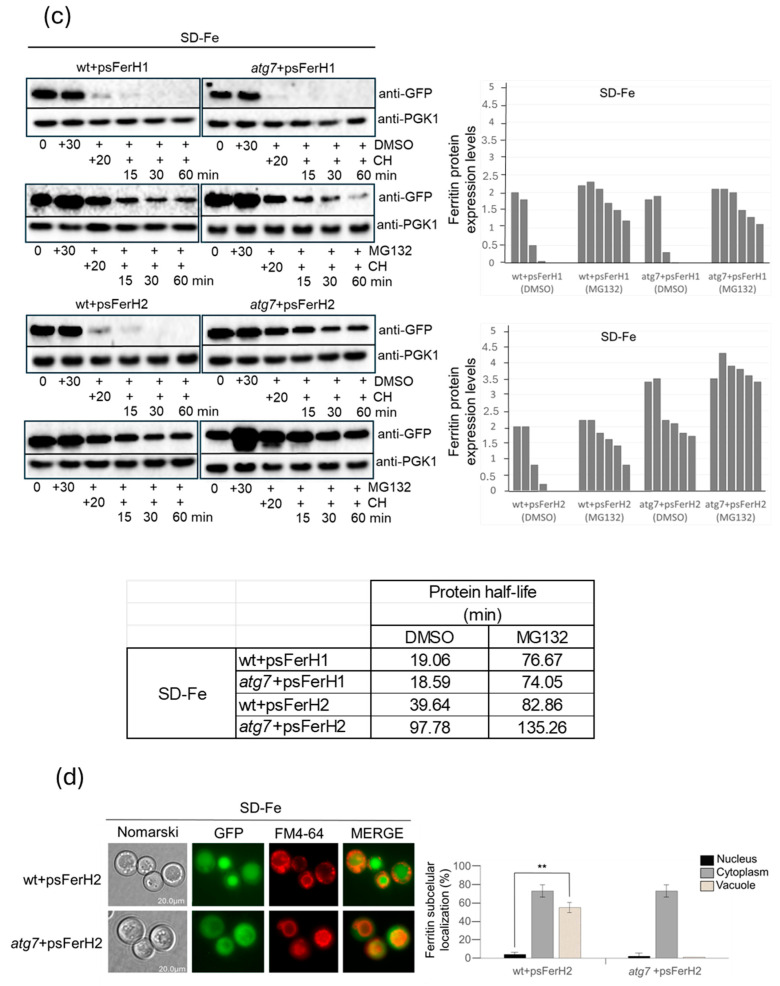
H2 soya bean ferritin is degraded in autophagosomes and proteasomes when iron is scarce. (**a**) Wild-type and *atg7* strains expressing each of H or L ferritins were exponentially grown in SD medium minus iron to OD_600_: 0.6. Each culture was split into two, and one half was treated with 75 µM MG132 for 30 min to block proteasome, whereas only DMSO (reagent used to resuspend MG132) was added to the other half. Subsequently, CH was added to all of the cultures, and upon 20 min of incubation, it was considered time 0, as shown in Figure 2. Histograms represent levels of each ferritin, calculated as the ratio between the values determined with the anti-GFP antibody and those determined with anti-PGK1 as the loading control. (**b**) As in (**a**), however, wild-type and *atg7* strains were expressing H1 and H2 soya bean ferritins in the SD medium. (**c**) As in (**b**), however, the medium used was SD minus iron. (**d**) Wild-type and *atg7* strains expressing H2 soya bean ferritin were exponentially grown in SD-Fe medium. In vivo observation under a fluorescence microscope of H2 overexpressed fused to GFP. FM4-64 staining described in the Materials and Methods section was used to visualize vacuoles. Ferritin subcellular localization was recorded and plotted in histograms. Error bars in the histograms represent the standard deviation calculated from three independent experiments. The significance of the data was determined by *p*-values from a Student unpaired *t*-test denoted as follows: ** = 0.01 > *p* > 0.001. Original images of Figure 3 can be found in Supplementary Materials.

It has been described that iron deprivation provokes the release of iron from ferritin nanocages through two principal mechanisms: ferritin degradation mediated by proteasome and/or autophagy. In order to obtain deeper information regarding iron recycling from ferritins when expressed in budding yeast, we decided to explore whether iron recycling from these proteins occurred through proteasome and/or autophagy. To analyze the possible role of proteasome, we used its inhibitor MG132. We observed that proteasome inhibition did not cause any change in human ferritin stability, with respect to untreated samples neither in the SD medium nor in the SD medium depleted of iron (Figure 3a). However, we also obtained different information regarding soya bean ferritins. The addition of MG132 clearly provoked a remarkable extension in the half-life of both H1 and H2 soya bean ferritins, as compared to samples where proteasome was not inhibited, both in the presence of iron or in cultures starved for the metal (Figure 3b,c). However, iron starvation accelerated both H1 and H2 ferritin degradation through proteasome (Figure 3c). These results suggest that proteasome participates in the stability and release of iron from soya bean ferritins in a manner dependent on the content of iron in the nanocages. The high stability of human ferritins did not allow us to ascertain the intervention of proteasome in their degradation when expressed in budding yeast.

To determine the potential role that autophagy could play in iron recycling from ferritin nanocages upon deprivation of the metal, we used the mutant *atg7*, which is deficient in autophagy since *ATG7* is essential in the first steps of all types of canonical autophagy. Then, we performed stability assays in the presence or absence of iron. The results we obtained did not show any remarkable difference in the stability and expression of both human ferritins between the wt and *atg7* strains, both in the presence or absence of iron (Figure 3a). In conclusion, due to the enormous stability detected in human ferritins expressed in yeast cells, we were unable to work out the mechanisms of ferritin degradation in conditions of iron scarcity. However, we ruled out the possibility of bulk autophagy since we did not observe ferritins vacuolar localization in any of the conditions of study (Figure 1a).

When we performed stability assays in *atg7* strain growing in SD medium, expressing either H1 or H2 soya bean ferritins, we observed similar results to those described in wt cells, suggesting that H1 and H2 degradation was proteasome-dependent and autophagy-independent (Figure 3b). A similar conclusion was applied to H1 in iron starvation conditions (Figure 3c). However, in SD cultures deprived of iron, we detected a pronounced increment, both in the amount of H2 protein detected in the western blot and also in its stability in the *atg7* strain, as compared to the wt (Figure 3c). As stated above, the observation of soya bean ferritin localization indicated that only the H2 soya bean ferritin chain is localized to the vacuole upon iron starvation (Figure 1b). We checked this localization in the *atg7* strain and observed that in the absence of Atg7, H2 ferritin did not localize to the vacuole when iron was limiting (Figure 3d). This supports our hypothesis that Atg7 and, consequently, autophagy were mediating H2 vacuolar accumulation. Taking together these results, we can conclude that H2 soya bean ferritin is recycled in the vacuole through a mechanism of autophagy when iron availability is reduced. Furthermore, when we inhibited proteasome in these conditions, we observed an additional increment in H2 stability, suggesting that not only autophagy but at least in part proteasome both contribute to H2 ferritin degradation and iron recycling, as depicted in Figure 3c.

### 3.3. Both Human and Soya Bean Ferritins Protect Yeast Cells Against the Oxidative Effects Caused by H_2_O_2_ and Reduce the Impact of DSB in the DNA

**Figure 4:**
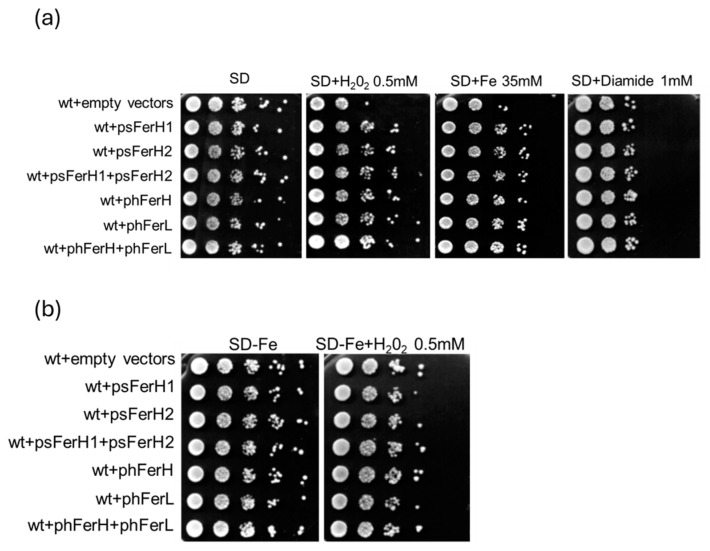
Effects of each of human or soya bean ferritin overexpression in wt viability upon the addition of oxidative agents. (**a**) Wild-type strain was transformed with pUG35 and pMM351 plasmids (empty vectors) or psFerH1, psFerH2, psFerH1+psFerH2, phFerH, phFerL, and phFerH+phFerL plasmids. Cultures were logarithmically grown (OD_600_:0.6) in SD medium plus amino acids at 30 °C, to be subsequently serial diluted and plated in triplicate onto SD plates containing or not the reagents: 0.5 mM H_2_O_2_, 35 mM Fe, and 1 mM diamide. Plates were grown at 30 °C for three days. (**b**) Cultures described in (**a**) were plated in triplicate onto SD-Fe plates containing or not 0.5 mM H_2_O_2_.

Fenton reaction is an important process that occurs in cells, and it is associated with the iron oxidative state in contact with hydrogen peroxide, giving rise to the accumulation of hydroxyl radicals, which are highly oxidative to the cell. We could observe that each of the four ferritins (human or soya bean) equivalently conferred wild-type cells with protection against the hydroxyl radical produced by the Fenton reaction in plates containing either iron or the oxidizing agent hydrogen peroxide, as depicted in Figure 4a. In support of the hypothesis that ferritins protect against oxidation caused by iron, through the Fenton reaction, we observed that neither human nor soya bean ferritins rescued the effects in cell lethality caused by hydrogen peroxide in plates limited for iron (Figure 4b). We also used diamide, another oxidative agent that is not involved in the Fenton reaction (it causes the formation of disulphide bonds but does not affect ROS accumulation in cells). In this case, ferritins did not rescue the effects of cell lethality caused by diamide (Figure 4a).

**Figure 5:**
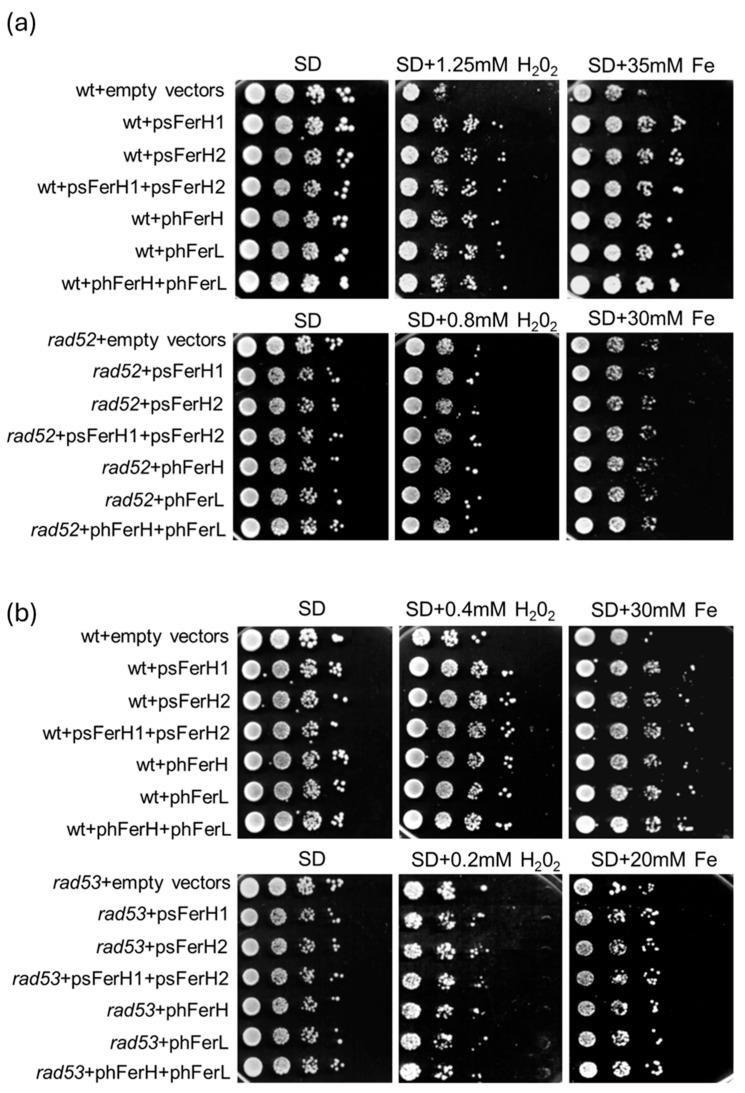
Wild-type resistance to H_2_O_2_ conferred by each human or soya bean ferritin overexpression is dependent on Rad53 expression. (**a**) Wt and *rad52* strains were transformed with pUG35 and pMM351 plasmids (empty vectors) or psFerH1, psFerH2, psFerH1+psFerH2, phFerH, phFerL, and phFerH+phFerL plasmids. Cultures were logarithmically grown (OD_600_:0.6) in SD medium plus amino acids at 30 °C, to be subsequently serial diluted and plated in triplicate onto SD plates containing or not different concentrations of H_2_O_2_ and Fe. (**b**) As in (**a**), however, wt and *rad53* strains were used. Plates were grown at 30 °C for three days.

Once we demonstrated the important role that ferritins play in the defense against hydroxyl radicals in a manner dependent on iron, we decided to investigate how this protection affects DNA damage caused by ROS. Hydrogen peroxide provokes both single and double-strand breaks, and as explained above, excess iron is also toxic due to the induction of the Fenton reaction. We decided to check the importance of ferritins in the protection against DSB damage caused by either hydrogen peroxide or iron overload. Rad52 is a protein involved in DSB repair upon DNA damage, whereas Rad53 is a protein that receives the signal of DNA damage and becomes activated through phosphorylation to induce cell checkpoint responses to repair the damage. In Figure 5a,b it is shown that the capability of either human or soya bean ferritins to protect cells against ROS was significantly impaired in both *rad53* and *rad52* mutants since none of the ferritins were able to rescue the viability of both rad mutants in the presence of either hydrogen peroxide or iron excess. Given that *RAD53* is a DNA damage checkpoint gene, our interpretation of the former results was that both iron and hydrogen peroxide caused DNA damage that at least in part provoked double-strand breaks for which repaired *RAD52* was necessary since it is known to be essential for DSB repair and homologous recombination in budding yeast. 

**Figure 6:**
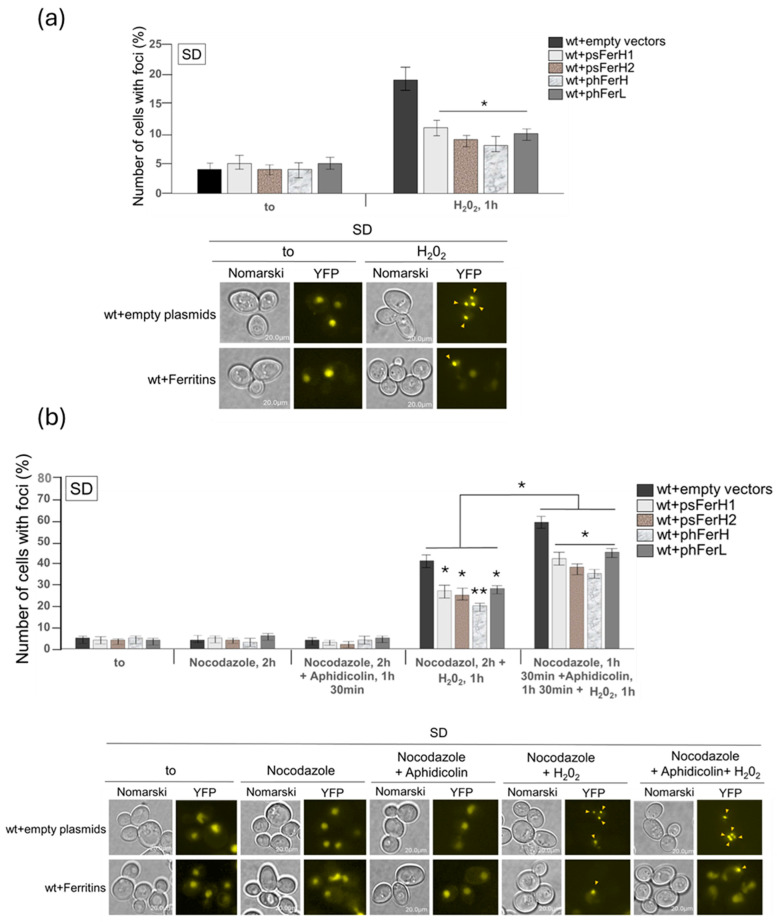

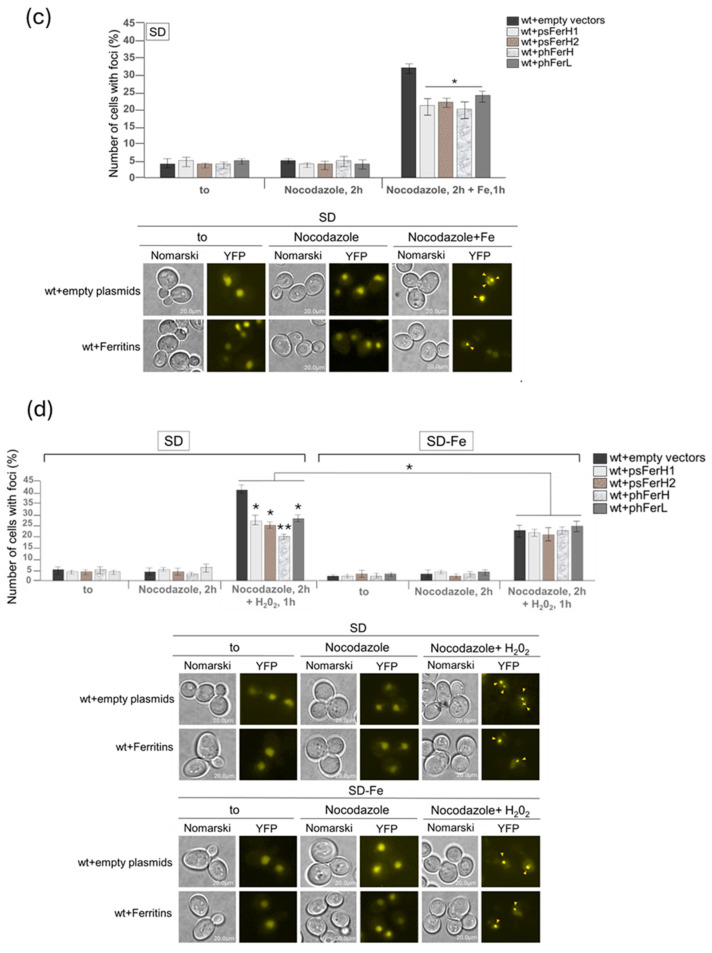
The four human and soya bean ferritins reduce the number of DSB upon wild-type treatment with H_2_O_2_ or iron excess. Wild-type cells expressing each of H1, H2 H, or L ferritins were transformed with a centromeric plasmid expressing RAD52YFP under its own promoter and grown in SD medium at 30 °C to perform different treatments with the aim to determine Rad52 DSB foci in the fluorescence microscope. (**a**) Rad52-YFP foci accumulation in asynchronous cultures of each of the strains, treated or not with 1 mM H_2_O_2_ for one hour. (**b**) All the strains were synchronized in G2/M with 15 µgr/mL nocodazole for two hours, ¼ of each of the cultures were used as controls, ¼ was released in medium containing 1 mM H_2_O_2_ for 1 h, and the other ½ was released in SD medium containing 20 µgr/mL aphidicolin for 1 h and a half, then split into two halves, one half was used as a control and 1 mM H_2_O_2_ was added to the other half for another hour. (**c**) As in (**b**), each of the strains was grown in SD and synchronized in G2 with nocodazole for 2 h, half of the culture was used as control and 8 mM iron was added to the other half for one hour. We present statistical data corresponding to the experimental observation in the fluorescence microscope. To illustrate them, we only show images corresponding to wt+phFerH as a representative example since equivalent qualitative results were obtained upon the expression of the other three ferritins. (**d**) Each of the strains was grown in both SD or SD-Fe media and synchronized in G2 with nocodazole for 2 h. Half of the cultures were used as controls, and the other half were added with 1 mM H_2_O_2_ for 1 h. Error bars in the histograms represent the standard deviation calculated from three independent experiments. The significance of the data was determined by *p*-values from a Student unpaired *t*-test denoted as follows: * = 0.05 > *p* > 0.01; ** = 0.01 > *p* > 0.001.

To more profoundly study the potential role that Rad52 could be playing in DNA repair upon H_2_O_2_ in the presence or absence of the ferritins, we analyzed the formation of Rad52 DNA foci, which represent sites to where Rad52 is recruited to actively repair DSB lesions in the DNA. However, the four ferritins significantly reduced the number of Rad52 foci in exponentially growing cells upon the addition of H_2_O_2_ (Figure 6a). We next synchronized cells in G2 with nocodazole to eliminate the G0/G1 population since it has been previously described that in this phase of the cell cycle, the accumulation of Rad52 foci is much lower than in S or G2/M phases of the cell cycle [35]. Upon this synchronization, we could detect a higher increment in the percentage of cells with foci in response to H_2_O_2_ treatment in wild-type cells not expressing ferritins, meaning that this synchronization allowed us to detect a higher number of double-strand breaks whose repair was mediated by Rad52 (Figure 6a,b). Also, in this condition, we could also determine a higher reduction in foci formation upon overexpression of either H, L, H1, or H2 ferritin chains, as compared with wt cells not expressing these proteins (Figure 6b). The most efficient of the four ferritins in reducing DSB repair foci was the H human subunit.

In view of the former results, we wondered whether ferritins had an effect against DSB formation due to their function in protecting cells from the oxidative effects provoked by free iron or, alternatively, whether these proteins had a role in DNA DSB repair upon DNA oxidative damage independent of iron. In order to circumvent these questions, we decided to treat cells with aphidicolin, a drug known to block the DNA polymerase function required to repair DSB, along with Rad52. Our reasoning was that in the case that ferritins played a role in repair rather than in protection by sequestering Fe^3+^, the blockade in DNA polymerase activity and subsequent hydrogen peroxide treatment would cause several newly formed Rad52 foci in wt cells. In this context, the prediction would be that ferritin expression would not have any different effect on DSB and foci formation than the observed in cells not containing ferritins. Upon aphidicolin treatment and subsequent H_2_O_2_ addition, we observed a clear and equivalent increase in the number of Rad52 foci in all samples as compared to cells not treated with aphidicolin, suggesting that during the hour of duration of hydrogen peroxide treatment, DNA polymerase function when not blocked, induced a similar level of DNA repair in all the strains, independently on ferritins expression (Figure 6b). We also observed that upon aphidicolin treatment, the ratio in the number of Rad52 foci, determined in wild-type cells expressing ferritins versus non-expressing, was conserved and equivalent to that determined in former experiments not treated with aphidicolin (Figure 6b). With these results, we believe that the effects that ferritins exert in DNA DSB are mainly protective.

Based on that conclusion, we next hypothesized that if the main function of ferritins is to store iron in the oxidized state Fe^3+^ and to protect cells against the oxidative effects that the metal provokes due to the Fenton reaction, iron starvation should cause a reduction in Rad52 foci upon hydrogen peroxide treatment in cells expressing either human or soya bean ferritins. In line with this reasoning, iron excess should exacerbate the formation of Rad52 DSB foci. To ascertain this, we repeated the experiment depicted in Figure 6b using media either with excess or deprived of iron. As indicated above in the text, we observed that iron excess caused an increase in the formation of Rad52 foci in wt cells with respect to cells cultured in SD control media (Figure 6a,c). Furthermore, overexpression of each of the four ferritins reduced significantly the accumulation of DSB foci in DNA regions (Figure 6c). However, neither human nor soya bean ferritin expression increased wt viability in plates deprived of iron in the presence of hydrogen peroxide (Figure 4b). Moreover, in these conditions, the accumulation of Rad52 foci experienced a significant reduction, as compared to cultures not deprived of iron and also based on the number of Rad52 foci that turned out to be equivalent between the empty control and cells expressing ferritins (Figure 6d). These results suggest that the effects that both hydrogen peroxide and iron overload cause in cell viability and DSB formation are mainly due to the iron stored in the cells and that the role that ferritins play in protecting cells against the noxious effect of ROS or iron excess are mainly due to their capability to oxidize and store iron thus avoiding the Fenton reaction.

### 3.4. Glycosylation Is Important in the Cellular Defense Against the Oxidative Effect Caused by ROS Dependent on Iron Carried Out by Both Human and Soya Bean Ferritins in the Budding Yeast Model

**Figure 7:**
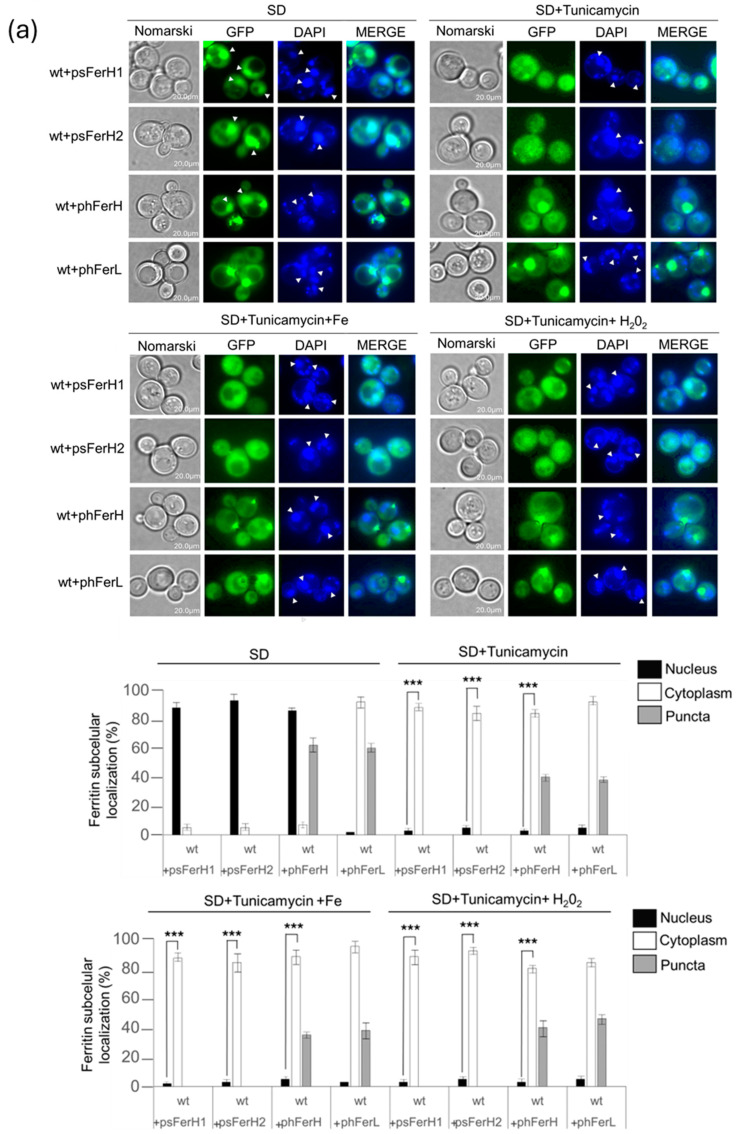

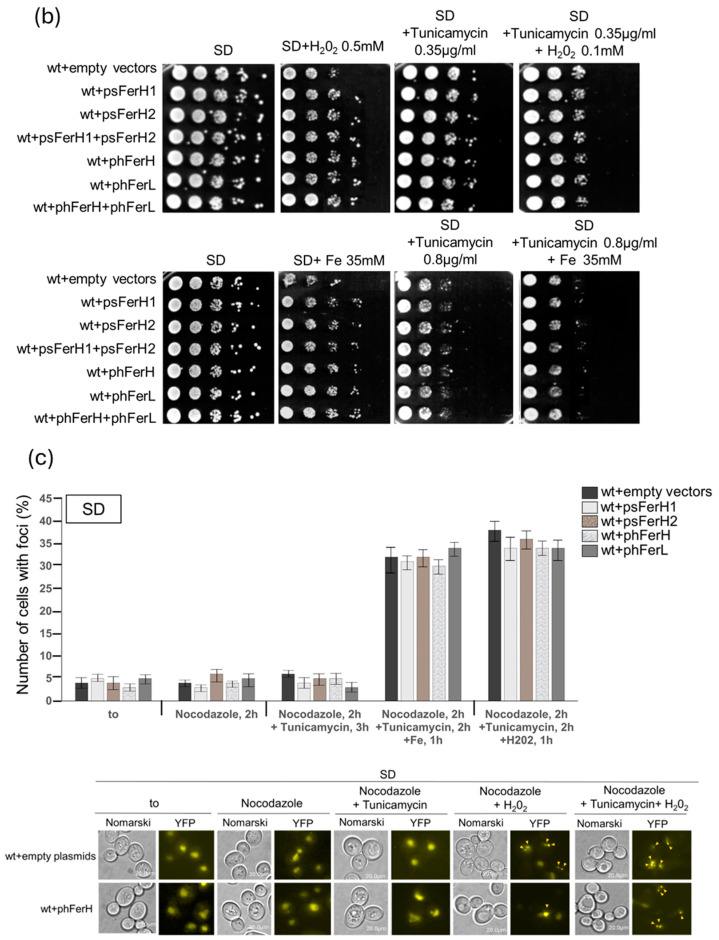
Glycosylation of the four H, L, H1, and H2 ferritins is important to maintain the protective function of these proteins against oxidative stress, which is dependent on iron. (**a**) Wild-type strain expressing either H1 and H2 soya bean ferritins or L or H human ferritins were grown to exponential phase (OD_600_ = 0.6) at 30 °C, in SD medium and cultures were split into four. One part was dedicated as control, and the other three parts were treated with 2 µgr/mL tunicamycin for two hours. Again, one of these three parts was separated as control, and the other was treated with 1 mM H_2_O_2_ for one hour, whereas 8 mM Fe was added to the third part for one hour. Error bars in the histograms represent the standard deviation calculated from three independent experiments. The significance of the data was determined by *p*-values from a Student unpaired *t*-test denoted as follows: *** = 0.001 > *p* > 0.0001. (**b**) Cultures of wild-type strain expressing each of pUG35 and pMM351 plasmids (+empty vector) or psFerH1, psFerH2, psFerH1+psFerH2, phFerH, phFerL, and phFerH+phFerL plasmids were grown to OD_600_:0.6 in SD medium plus amino acids at 30 °C, to be subsequently serial diluted and plated in triplicate onto SD plates containing or not the reagents: 0.5 mM H_2_O_2_, 0.35 µg/mL Tunicamycin, 0.35 µg/mL Tunicamycin+0.1 mM H_2_O_2_, 35 mM Fe, 0.8 µg/mL Tunicamycin and 0.8 µg/mL Tunicamycin+35 mM Fe. Plates were grown at 30 °C for three days. (**c**) All the strains described in Figure 6 were synchronized in G2/M with 15 µg/mL nocodazole for two hours, ¼ of each culture was used as control, ¼ was treated with 2 µg/mL Tunicamycin for three hours as control, ¼ was treated with 2 µg/mL Tunicamycin for two hours and, subsequently, iron was added at 8 mM final concentration for one hour and the last ¼ was treated with 2 µg/mL Tunicamycin for two hours then 1 mM hydrogen peroxide for 1 h. We present statistical data corresponding to the experimental observation in the fluorescence microscope. We only show images corresponding to wt+phFerH as a representative example since equivalent qualitative results were obtained upon the expression of the other three ferritins. Error bars in the histograms represent the standard deviation (SD) calculated from three independent experiments. The significance of the data was determined by *p*-values from a Student unpaired *t*-test denoted as follows: *** = 0.001 > *p* > 0.0001.

Human ferritins are known to be post-translationally modified, being glycosylated [9]. We decided to test whether either human or soya bean ferritins experienced any functional changes when the process of glycosylation was impaired in budding yeast. Since inhibitors of the O-glycosylation have been proposed to prevent the nuclear translocation of human H ferritin to the nuclei [9], we made use of the double mutant *pmt1pmt2*, which is impaired in O-glycosylation, in order to check whether H, H1, and H2 chains were affected in their nuclear localization. However, we did not detect any change with respect to what we have previously observed in wt cells. We next used the drug tunicamycin, which blocks N-linked glycosylation, and treated cell cultures with either high iron concentrations or hydrogen peroxide. We observed that N-linked glycosylation blockade provoked nuclear ferritin (H, H1, and H2) to translocate to the cytoplasm independently on the subsequent treatments (Figure 7a). Moreover, when N-linked glycosylation was blocked, the ability of the four ferritins to rescue viability when iron was overloaded in the media or upon peroxide treatment was impaired (Figure 7b). We also analyzed the capacity of ferritins to reduce the number of Rad52 foci caused by either excess of iron or hydrogen peroxide in the presence of tunicamycin and observed that the reduction was significantly impaired as compared with cells not treated with tunicamycin (Figure 7c). These observations were supported by our unpublished results obtained with the thermosensitive mutant *sec18-1*, defective in secretion and N-linked glycosylation. Our observations suggest that impairment of N-glycosylation alters the nuclear localization of human H and soya bean H1 and H2 ferritins when overexpressed in budding yeast and also negatively affects the function of the four human H and L and soya bean H1 and H2 ferritins in cell protection against the negative effects that iron overload and the subsequent oxidative stress could cause in cells viability and DNA damage.

## 4. Discussion

The yeast *Saccharomyces cerevisiae* has been widely and successfully used as a model organism to express heterologous proteins from high eukaryotes in order to study different related disorders. This is due to several reasons, such as the well-known genetics, metabolism, and physiology, and the easy manipulation of the yeast genome. Here, we use this model to express either human or soya bean ferritins with the aim of identifying new functions and validating this approach for potential future beneficial use in human health research. Despite the fact that chimaeras presented in this study might differ somehow from the native ferritins, in particular soya bean chains, we consider that our results will positively contribute to the knowledge of these proteins, as we discuss below.

The results reported here reveal that both soya bean and human ferritins, when heterologously expressed in the eukaryotic model *S. cerevisiae*, display common functions related to the protection against oxidative stress—which is dependent on the Fenton reaction—and also against the harmful consequences in DNA damage, in particular in double-strand breaks.

The high stability of human ferritins is a characteristic previously observed in human cells [36,37], and our results confirmed that this characteristic was conserved when humanized in *Saccharomyces cerevisiae* [36] and this study. Our results are also in accordance with [36]: human ferritins expressed in budding yeast do not show the lysosomal degradation observed in human systems. These authors observed a partial degradation of human ferritins through proteosomes over long periods of time. Our data demonstrate that both H and L human ferritins show a high stability of more than 4 h when overexpressed in budding yeast. In our culture conditions, we were unable to significantly determine the participation of proteasomes in their degradation. We hypothesize that the discrepancy could be due to several circumstances; one is that unlike [36], we do not conduct our assays in the *erg6* mutant, and another is that both our yeast background and the regulatable promoter are different from those used in the studies published by [36]. More differences are that we blocked protein synthesis with cycloheximide, which is not stable upon 7 h, and that we used conditions of iron starvation.

It has been reported that plant ferritin stability depends on a potential cross-talk between the vacuole and plastids, which are the compartments that accumulate iron stored in ferritins, as demonstrated in *Arabidopsis* seeds [38].

Here we show a novel observation for soya bean ferritins, which is the degradation through proteasome when iron is scarce as a mechanism to liberate and recycle iron, and the participation of bulk autophagy, as the main mechanisms in charge of that function only in the case of H2 ferritin. Interestingly, in mammals, only H ferritin has been detected to bind NCOA4 in the process of ferritinophagy [39].

Our results indicate that soya bean ferritins are much less stable than their human counterparts when overexpressed in yeast. Interestingly, our results are coincidental with the observations that [40] made with pea ferritins, whose stability was as long as 15 min at pH between 2 and 4, as opposed to the high stability and acid–base tolerance that human ferritins present [41,42]. Furthermore, we present evidence demonstrating that the H2 soya bean subunit is more stable than H1 in a manner dependent on both the excess and the absence of iron (Figure 3b,c). This again supports previous observations made by [43], in which they establish that the H-2 subunit of plant ferritin is more resistant to proteolysis than the H-1 subunit.

Human ferritin localization was initially considered to be cytosolic as iron storage. However, it has been linked to function when they are located in different compartments such as mitochondria or nuclei [44]. Here, we demonstrate that the localization of human ferritins in *S. cerevisiae* turned out to be similar to that observed in liver and spleen cells, where H is partly localized in the nucleus, and L is predominantly found in the cytosol [45]. In plant models [46], ferritins have been detected in plastids and mitochondria. Plastids are not present in yeast or in human cells. However, in the absence of plastids, both H1 and H2 soya bean ferritins do not localize to mitochondria but mainly in the nucleus, as occurs with the H human chain. More intriguing and interesting is the fact that both soya bean ferritins, being nuclear, are functional in a manner similar to both H and L subunits. Our results also suggest that N-glycosylation affects H human and H1 and H2 soya bean ferritins since blockade with tunicamycin prevents nuclear localization in all of them indistinctly.

One accepted function for human ferritins, based on numerous reports, is that they both play a cytoprotective role against intracellular oxidative damage [11,18]. Although some other reports [47,48] grant the H subunit a more protective function than ferritin L, in our model, both H and L human proteins, as well as H1 and H2 soya bean ferritins, show similar ability to protect cells against oxidative damage. Furthermore, our model allowed us to determine that either human or soya bean ferritins are similarly functional in the rescue of the oxidative stress that is specifically linked to the presence of iron, such as that provoked by H_2_O_2_, since we show that when oxidative damage is not dependent on iron, as is the case of diamide, this rescue is not detected (Figure 4a,b).

On the other hand, human ferritins have been associated with iron storage and oxidative protection in bacteria, whereas in plants, there are many reports that suggest a high variation depending on the vegetable and the different parts of the specific plant. However, roles in iron storage and oxidative stress protection have been reported (for a review, [49]). Thus, in the vegetal model *Arabidopsis thaliana*, ferritins play a significant role in the defense against pathogens and oxidative stress [49,50]. In a previous study [51], the authors show that ectopic expression of plant ferritins favored more tolerance to oxidative stress. Rice ferritins have been suggested to accumulate in vacuoles to buffer rice plants against Fe overload and oxidative damage [49,52]. In this study, we present results demonstrating that soya bean ferritin overexpression in the heterologous eukaryotic system lacking plastids causes a higher resistance to oxidative stress provoked by H_2_O_2_ and iron overload. This observation extends the universal effect that ferritins have, no matter the localization or maturation in specific organelles, or alternatively, suggests that heterologous expression is perfectly functional no matter the animal or vegetal origin of these proteins.

ROS, such as superoxide radical, H_2_O_2,_ or hydroxyl radical, can provoke DNA damage, including single or double-stranded DNA breaks in prokaryotes and lower eukaryotes [53], although identical effects have been described in human organs such as thyroid [54]. In *S. cerevisiae*, [55] observed that exogenous H_2_O_2_ induced an increase in Rad52 foci, which are sites of Rad52-dependent recombination and DSB repair [24]. In epithelial mammalian cells, an increase in gamma H2AX foci (a marker of DSB, equivalent to *S. cerevisiae* Rad52 foci) has been detected in conditions of iron overload upon irradiation [56]. Moreover, local iron depositions have been demonstrated to cause oxidative damage, including DNA double-stranded breaks [57]. Ferritin protection against DNA strand breaks caused by an iron-containing environment and Fenton reaction have been shown by [22]. Other reports also link human ferritins to DNA damage dependent on iron. Thus, malignant mesothelioma has been reported to be associated with mutant Brca1 haploinsufficiency and was related to Fe^2+^ accumulation and to the decrease in ferritin expression since BRCA1 is a first line of defense against DSB, and the consequence was the oxidative DNA damage [58].

Regarding plant ferritin’s function in protection against DNA damage, it has been reported that either soya bean or black bean ferritins can protect against plastid DNA damage from oxidative stress, although these conclusions were limited by the use of an in vitro assay [59,60]. Furthermore, in vitro assays described in the literature have revealed that bacteria and H human subunit ferritins are able to bind DNA [61]. The authors associated this ability with a function related to DNA nicking protection. However, they were not conclusive.

The results presented here clearly demonstrate that each of the human and soya bean ferritins is capable of protecting yeast DNA from DSB by reducing Rad52 foci formation upon treatment with iron overload or with oxidant agents, whose effects are associated with the presence of iron, as occurs with the use of exogenous H_2_O_2_.

Budding yeast can be a suitable model for studying conserved functions such as DSB repair, DNA damage checkpoints, and bulk autophagy. Our results also open the door to speculation of whether ferritin degradation or recycling might occur in different forms depending on the conditions. Recycling through the lysosome using or not the canonical autophagy mechanisms is generally accepted, in special conditions of iron excess, to avoid excess of iron storage and consequently reduced iron availability (reviewed in [62]). Nowadays, the finding of NCOA4 as a specific receptor for ferritinophagy and ferroptosis has put the focus on this process as a main one [63]. There is no homologous to NCOA4 in *S. cerevisiae* identified thus far. However, our results open the possibility that autophagy or general autophagy not necessarily dependent on NCOA4 might also play a role in the recycling of some ferritins like soya bean ferritin H2. This observation deserves more experimental data due to its important repercussions on human or plant health.

Plant ferritins have sites for ferritin glycosylation, as identified in rice by [64]. However, there are no reports regarding their function. Both L and H have glycosylation sites. However, the fact that the H protein has more numerous sites than L ferritin permitted [21] to conclude that H glycosylation, and more specifically, O-glycosylation, is important for its nuclear localization. In some reports, low levels of glycosylated form compared to non-glycosylated form are associated with several human diseases [44]. Some reports claim that L ferritin is secreted through the classical secretory pathway [65]. Whereas more recently, other authors reported that human ferritin is secreted into serum via extracellular vesicles (EVs) or the secretory autophagy pathway but not via the classical endoplasmic reticulum (ER)-to-Golgi secretion pathway [66]. However, the current opinion of researchers is that this mechanism requires further experimental data since it is yet poorly understood, and there are substantial variations related to the different cell types. Furthermore, recently in [67], authors concluded that glycosylated ferritins in *Tegillarca granosa* exhibited better antioxidant capacity. Other authors [68] have reported that glycosylation does not seem to be limiting ferritin translocation, suggesting that possibly some glycosylated protein, still unknown, might be responsible for ferritin translocation. These observations are in line with our results since, in this study, we observe that glycosylation is not exclusive to ferritin nuclear translocation. Moreover, and given that at this point we cannot demonstrate that the effects of tunicamycin directly block ferritins N-glycosylation, we can speculate that, alternatively, this drug may be affecting an unknown protein which N-glycosylation would be necessary for ferritins function in oxidative stress resistance and DSB prevention. Further research will be required to unravel the specific and relevant mechanistic function of ferritin glycosylation (Figure 7).

Our results support the utility of the use of *S. cerevisiae* as a heterologous model to express and extend the knowledge in the fields of human and plant ferritin metabolism and suggest the existence of specific evolutive functional conservation between human and vegetal ferritins.

## 5. Conclusions

Heterologous overexpression of either human or soya bean ferritins allowed to demonstrate a functional conservation of the four proteins regarding:

(a) DNA damage protection against the oxidative effects provoked by iron as a consequence of Fenton reaction. In this study we demonstrate that the four ferritin chains reduce the formation of DSB.
(b) Reduction of oxidative damage and cell lethality caused by H2O2 in a manner totally dependent on iron.

## Data Availability

The original contributions presented in this study are included in the article/Supplementary Materials. Further inquiries can be directed to the corresponding author(s).

## Supplementary Materials

The following supporting information can be downloaded at https://www.mdpi.com/article/10.3390/biom15030447/s1, Figure S1. Determination of ferritins stability under several conditions.
