# Supplementary material for: Heterologous Expression of Either Human or Soya Bean Ferritins in Budding Yeast Reveals Common Functions Protecting Against Oxidative Agents and Counteracting Double-Strand Break Accumulation
Source: Biomolecules. 2025 Mar 20;15(3):447. doi: 10.3390/biom15030447 (PMC11939973; doi:10.3390/biom15030447)
Supplement: Supplementary file 1 [file biomolecules-15-00447-s001.zip › biomolecules-3478388-supplementary.pdf]

Figure S1

a)

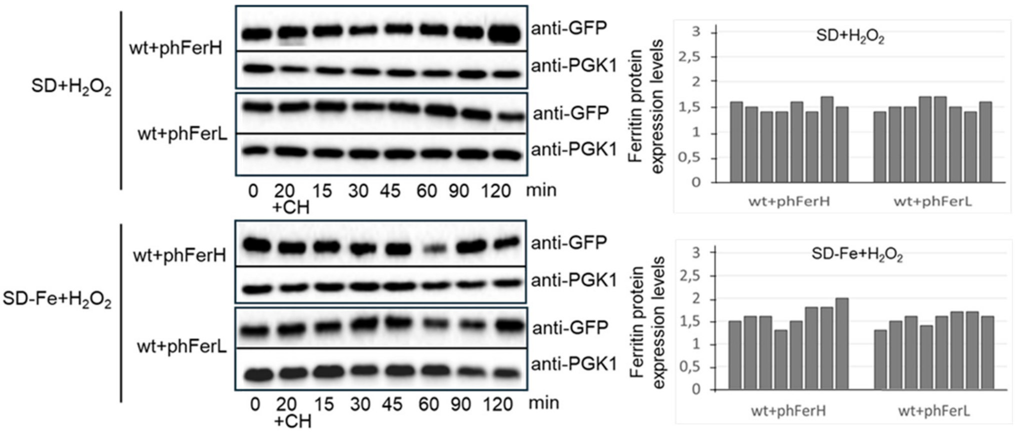

Figure S1

b)

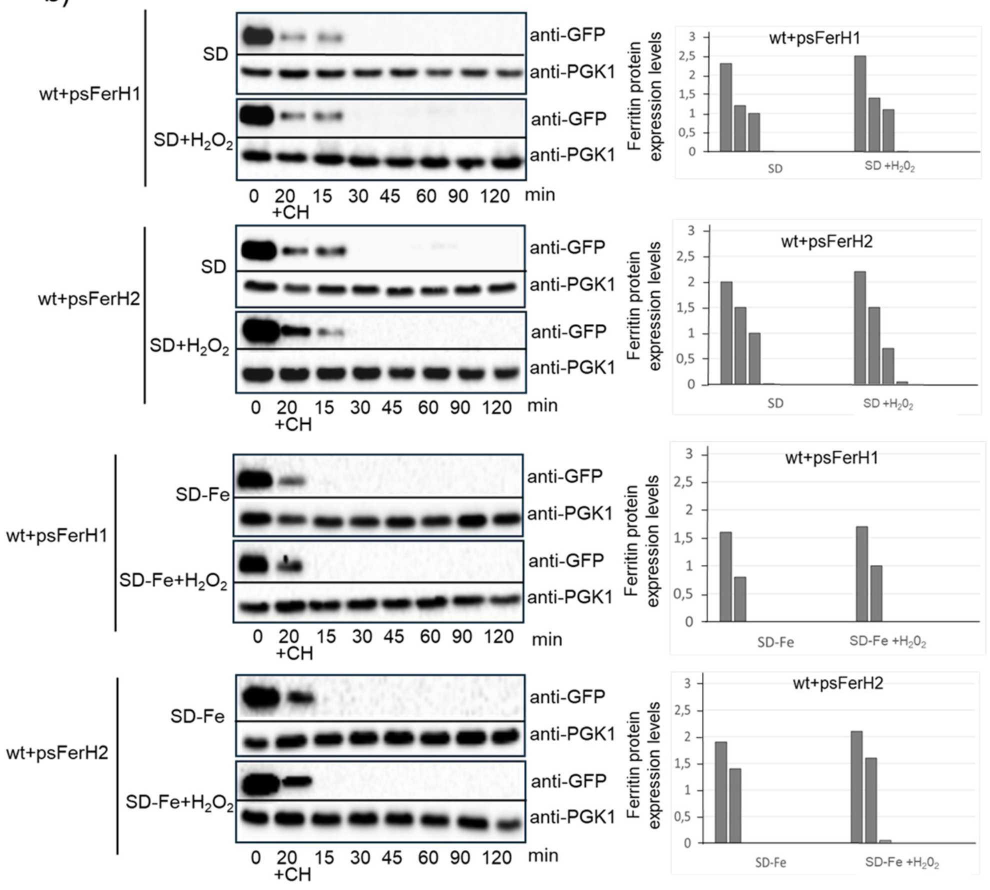

		Protein half-life (min)
SD	wt+psFerH1	31.42
	wt+psFerH2	33.34
SD+H_2O_2	wt+psFerH1	32.27
	wt+psFerH2	34.11
SD-Fe	wt+psFerH1	15.15
	wt+psFerH2	34.02
SD-Fe+H_2O_2	wt+psFerH1	15.62
	wt+psFerH2	34.21

Figure S1. **Determination of ferritins stability under several conditions**. a) Wild type cultures expressing each of H or L ferritins were exponentially grown in SD + 1mM H_2O_2 or SD-Fe + 1mM H_2O_2 to OD_{600}: 0.6. After that, all cultures were treated with 100µg/ml cycloheximide (+CH), 20 min of treatment was considered as time 0 for calculations. b) as in a) but wt was expressing either H1 or H2 soya bean ferritins. For all the western blot figures: Ferritin proteins were detected by using anti-GFP antibody whereas the loading control, Pgk1 was detected with anti-PGK1 antibody. Histograms represent levels of each ferritin calculated as the ratio between the values determined with the anti-GFP antibody with respect to those determined with anti-PGK1 as the loading control.
