# Supplementary material for: Heterologous Expression of Either Human or Soya Bean Ferritins in Budding Yeast Reveals Common Functions Protecting Against Oxidative Agents and Counteracting Double-Strand Break Accumulation
Source: Biomolecules. 2025 Mar 20;15(3):447. doi: 10.3390/biom15030447 (PMC11939973; doi:10.3390/biom15030447)

Figure 2
a)

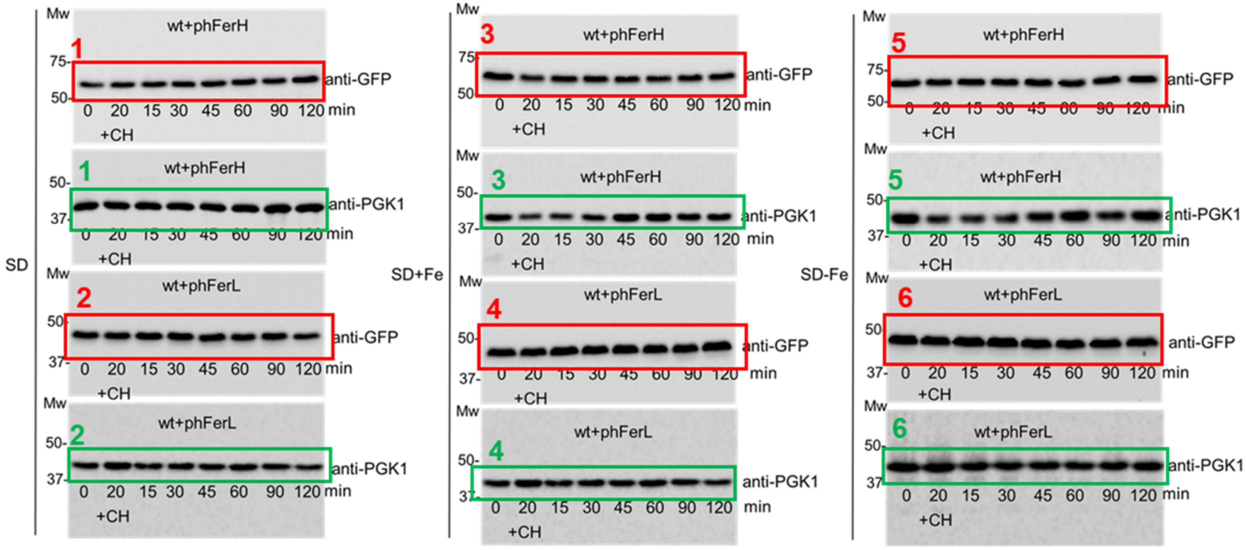

Figure 2

a)

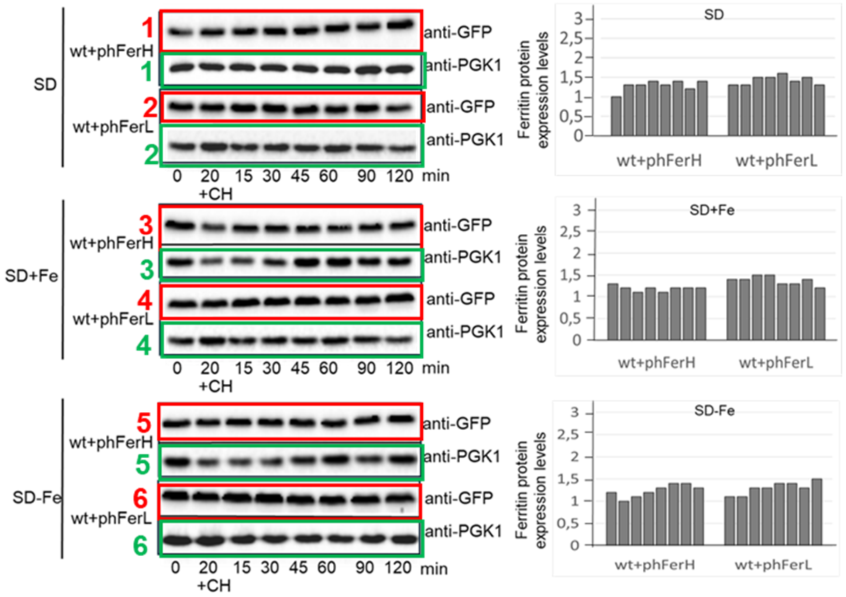

Figure 2
b)

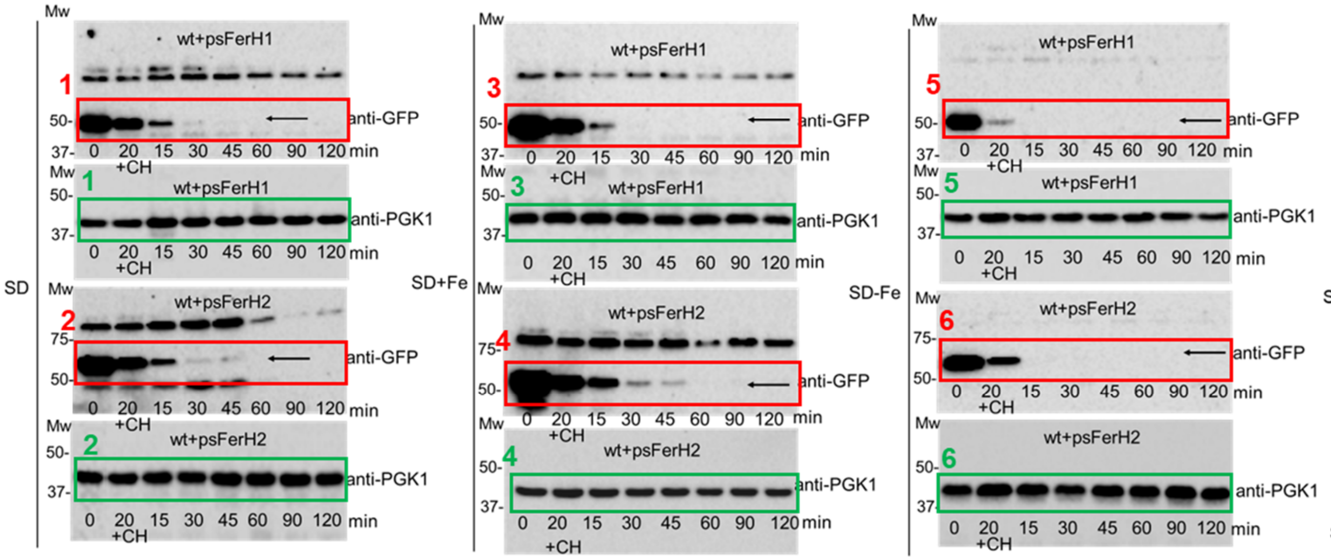

Figure 2

b)

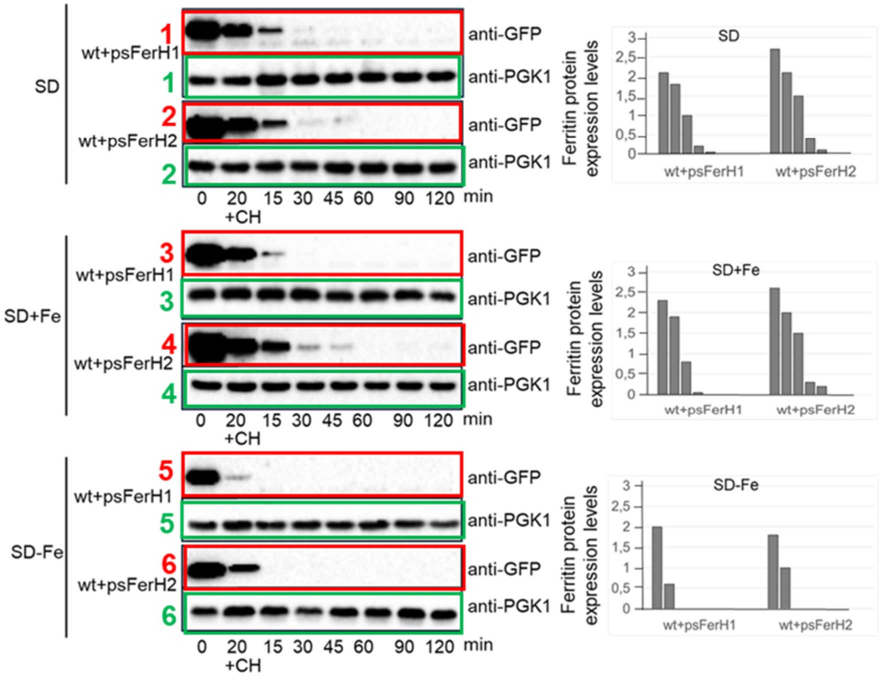

Figure 3

a)

Figure 3
a)

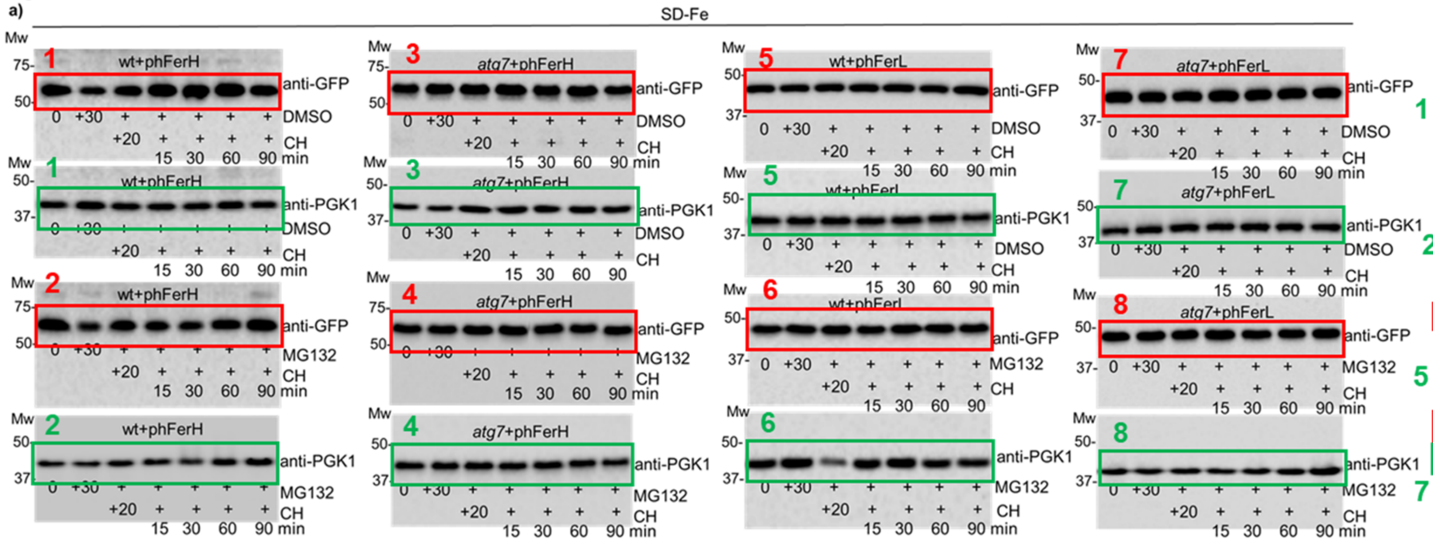

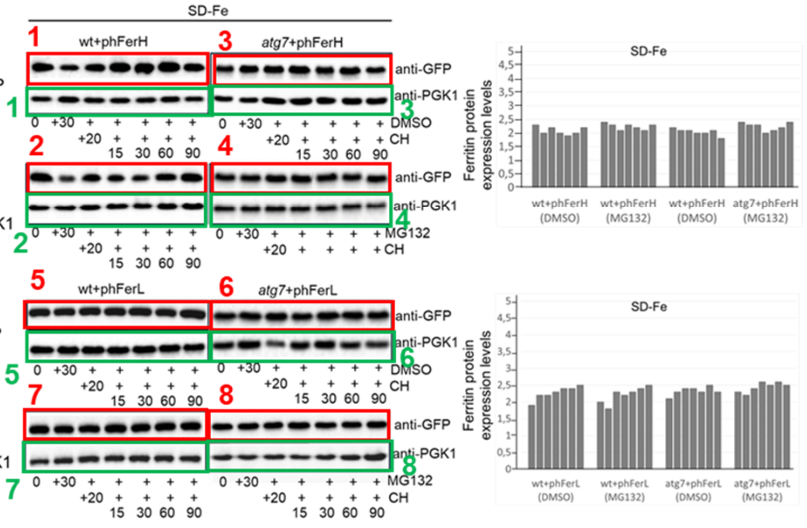

Figure 3

b)

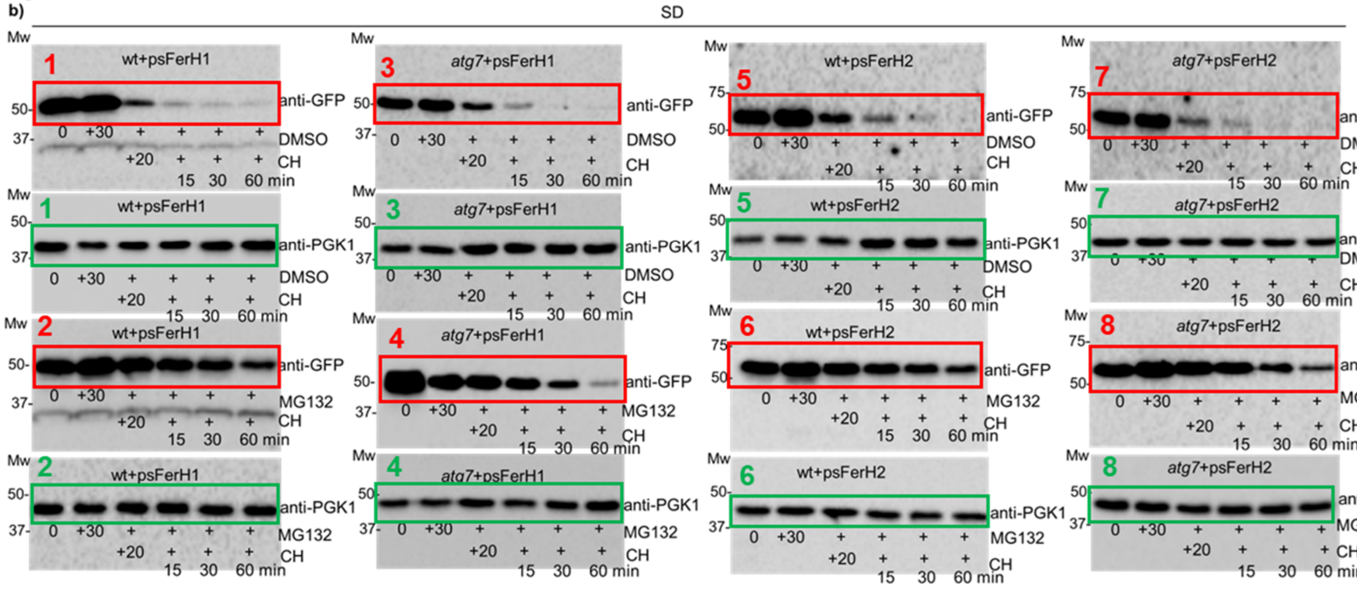

Figure 3

b)

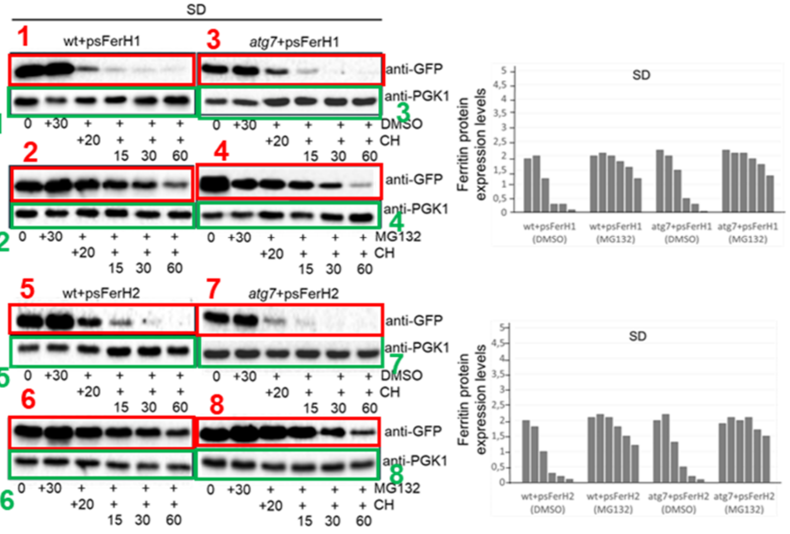

Figure 3
c)

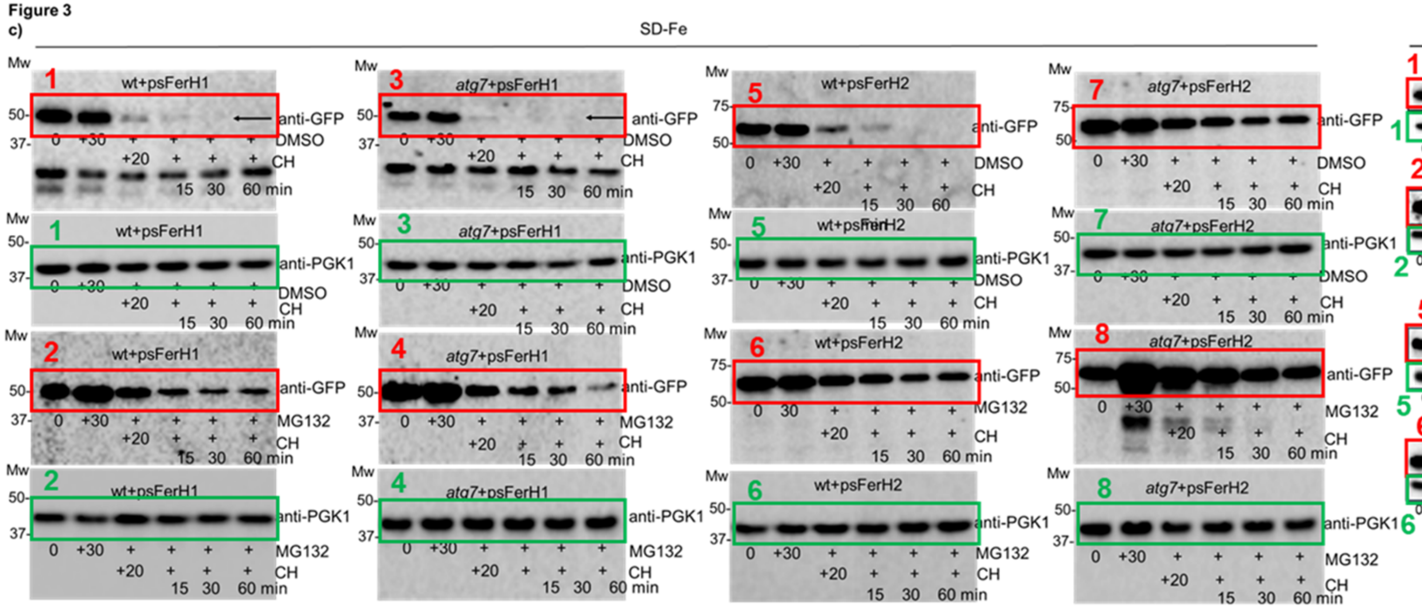

Figure 3

c)

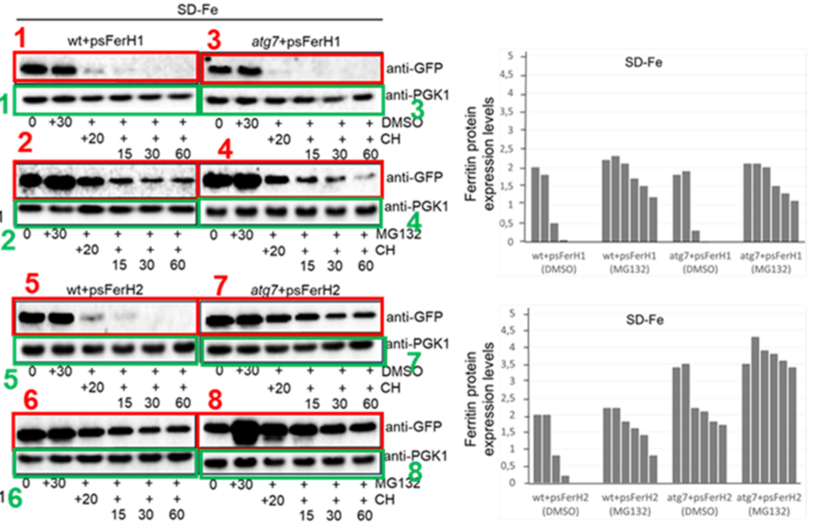

Supplementary Materials

Figure S1

a)

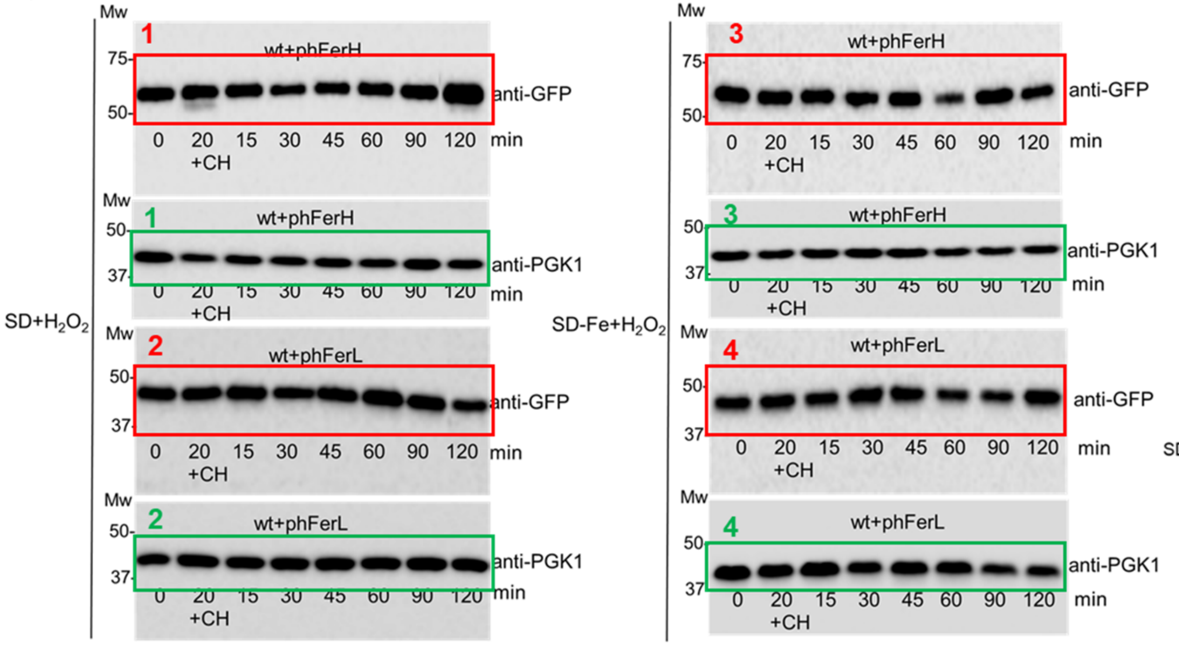

Figure S1

a)

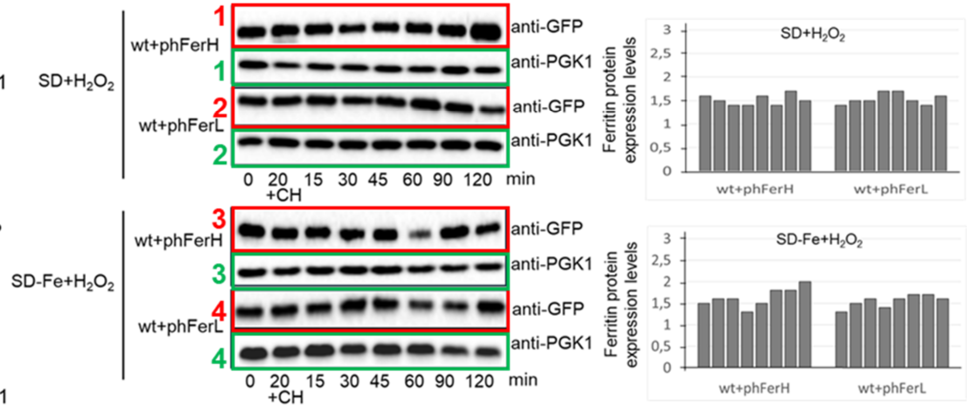

Supplementary Materials

Figure S1

b)

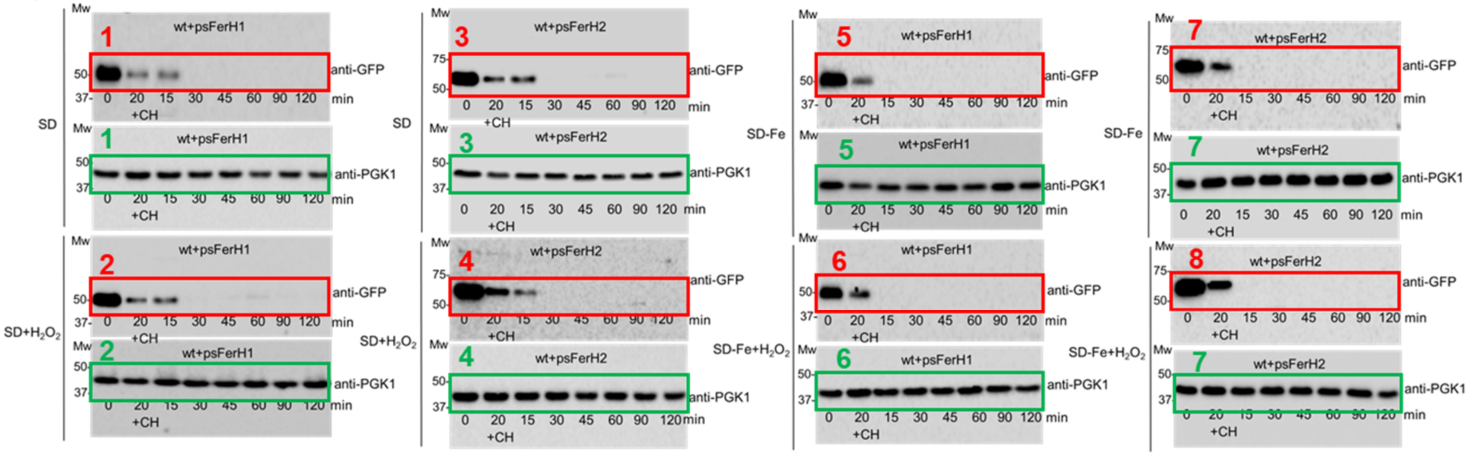

Figure S1

b)

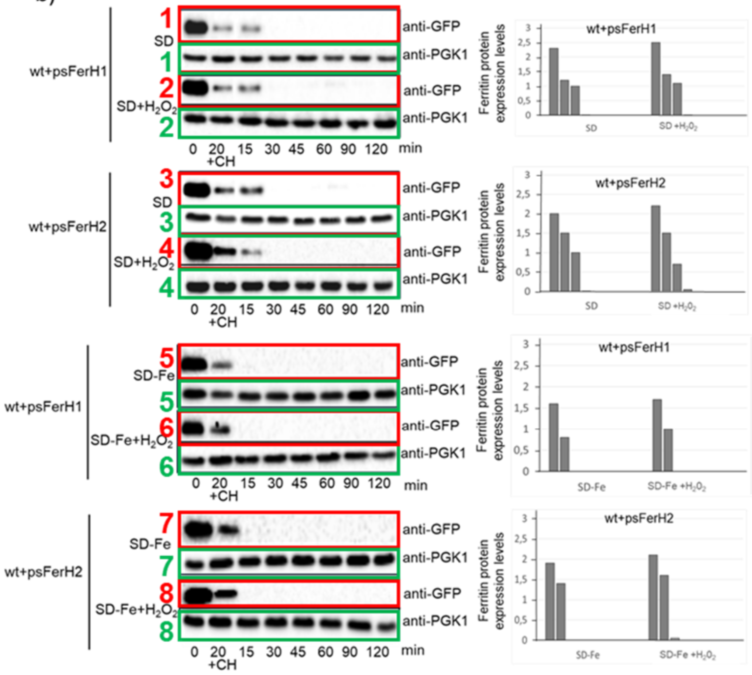

Figure 2
a)

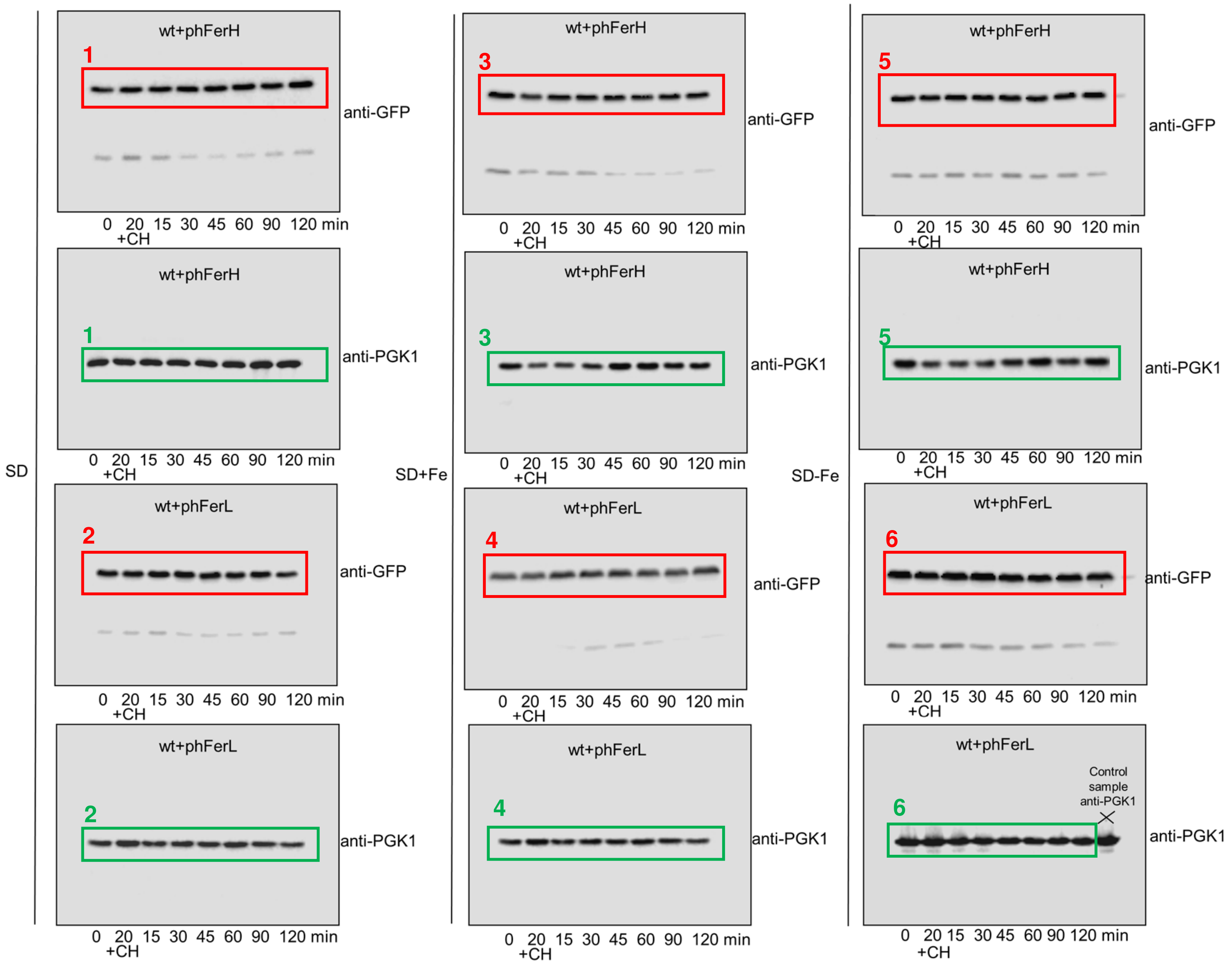

Figure 2
a)

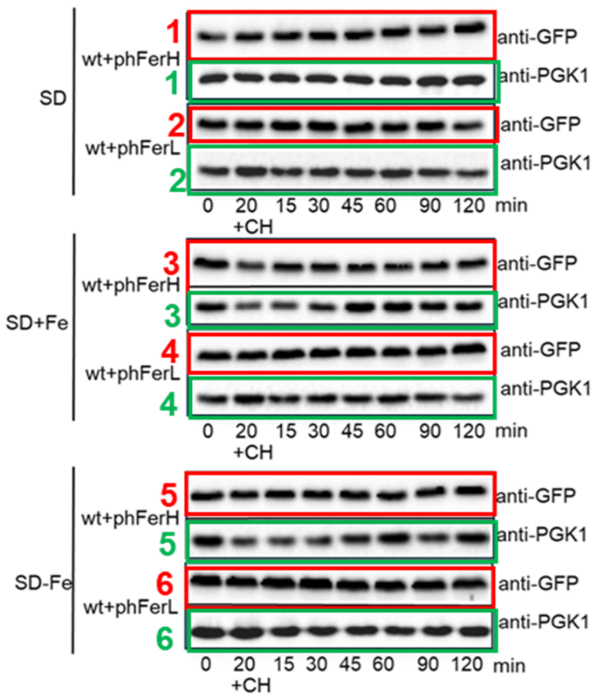

Figure 2
b)

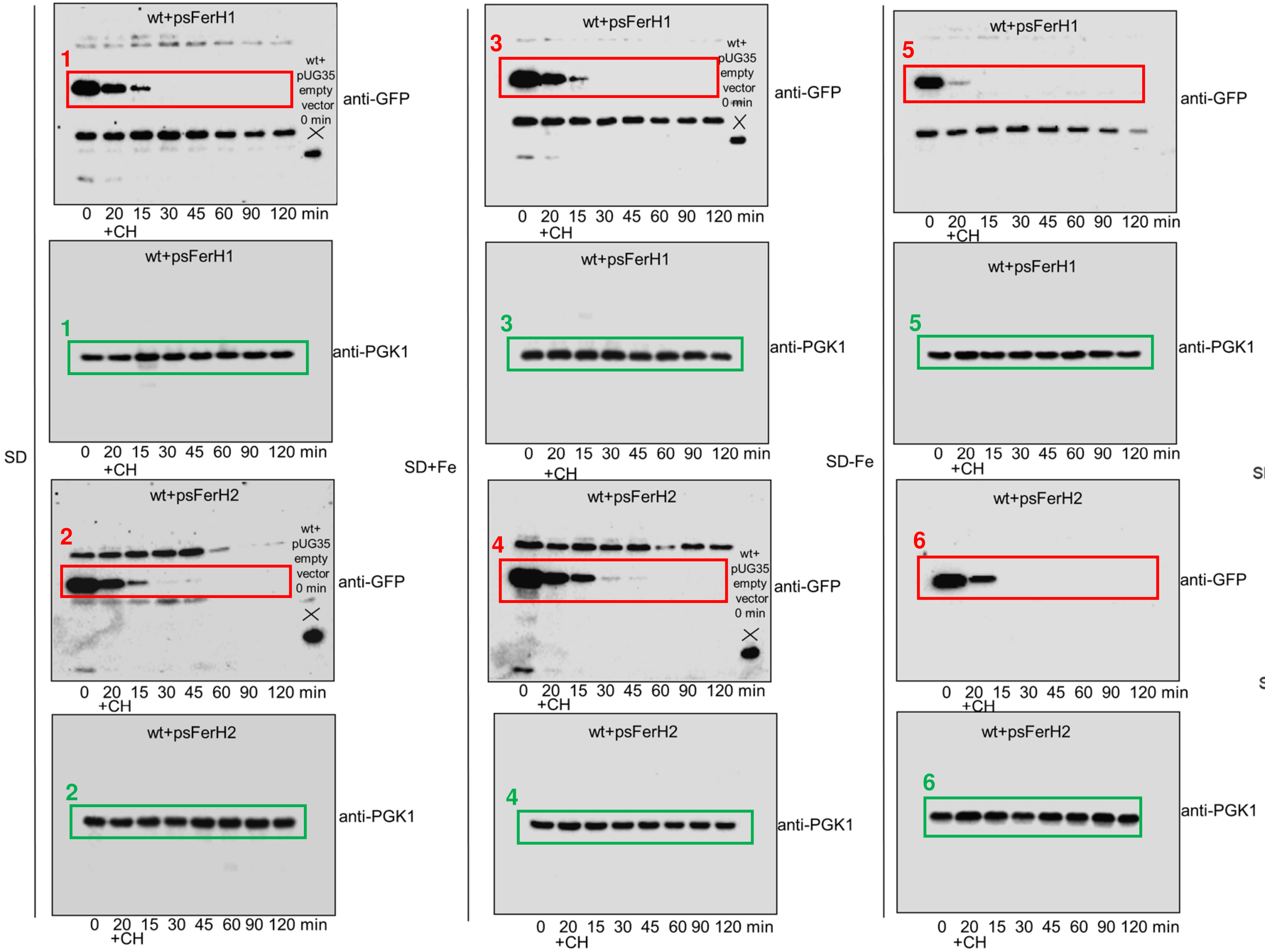

Figure 2

b)

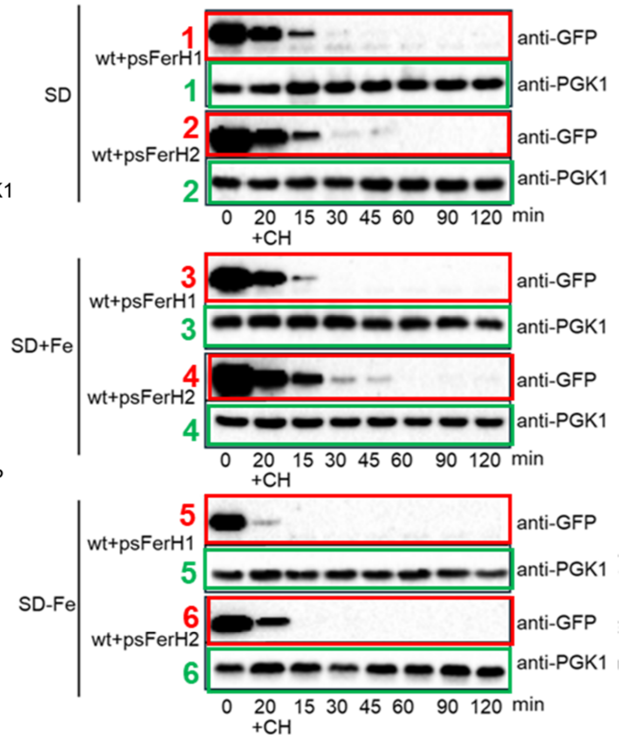

Figure 3
a)

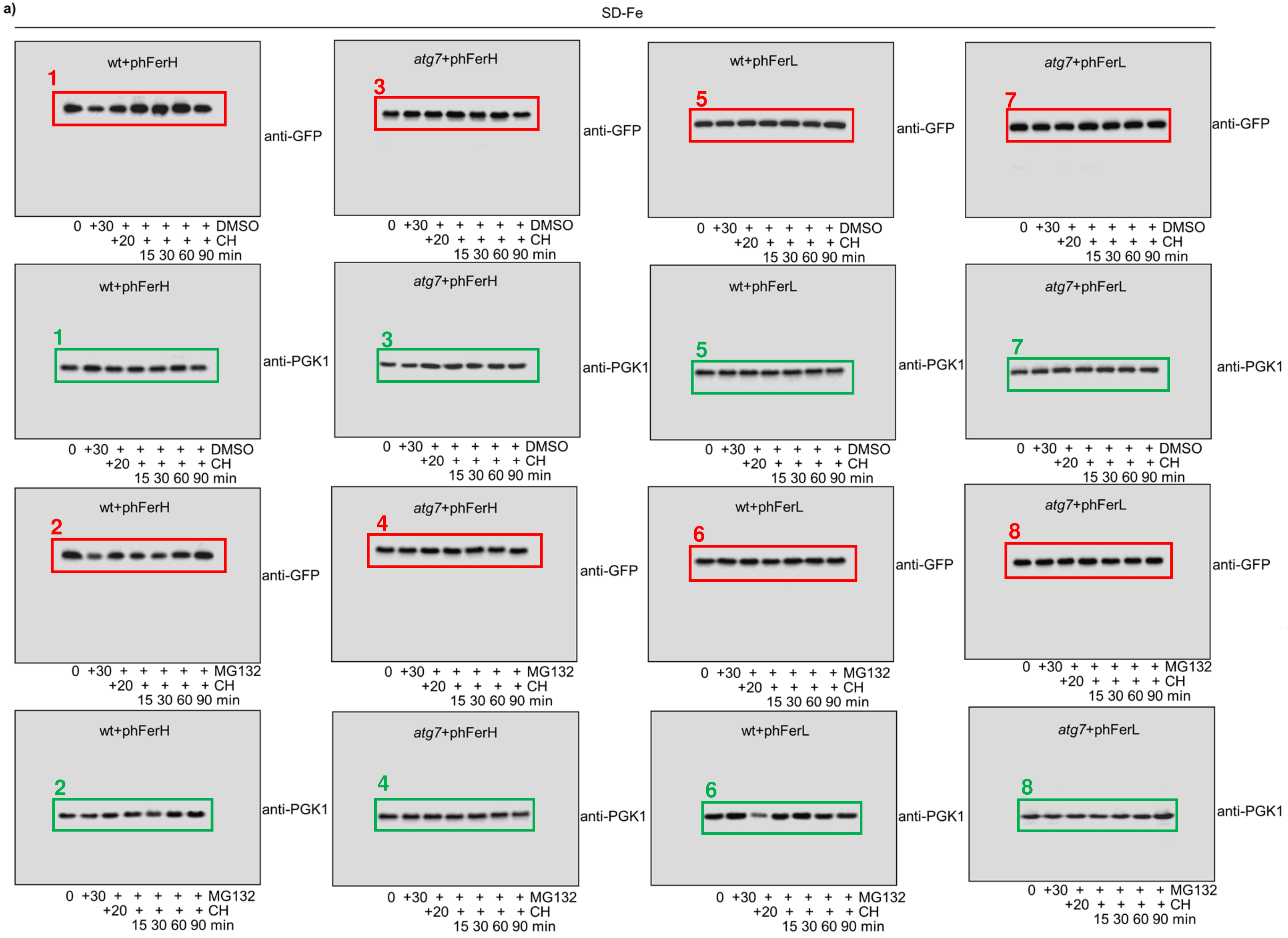

Figure 3

a)

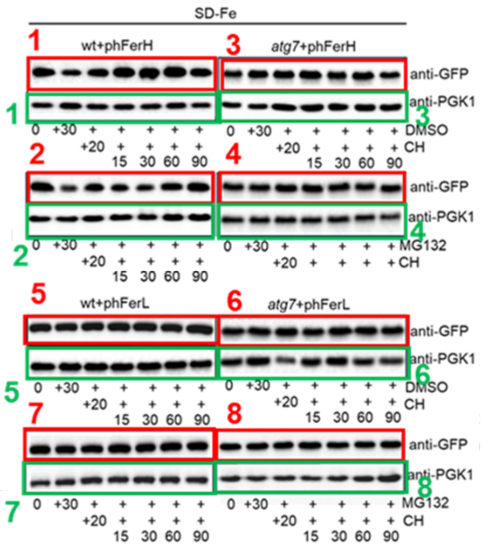

Figure 3
b)

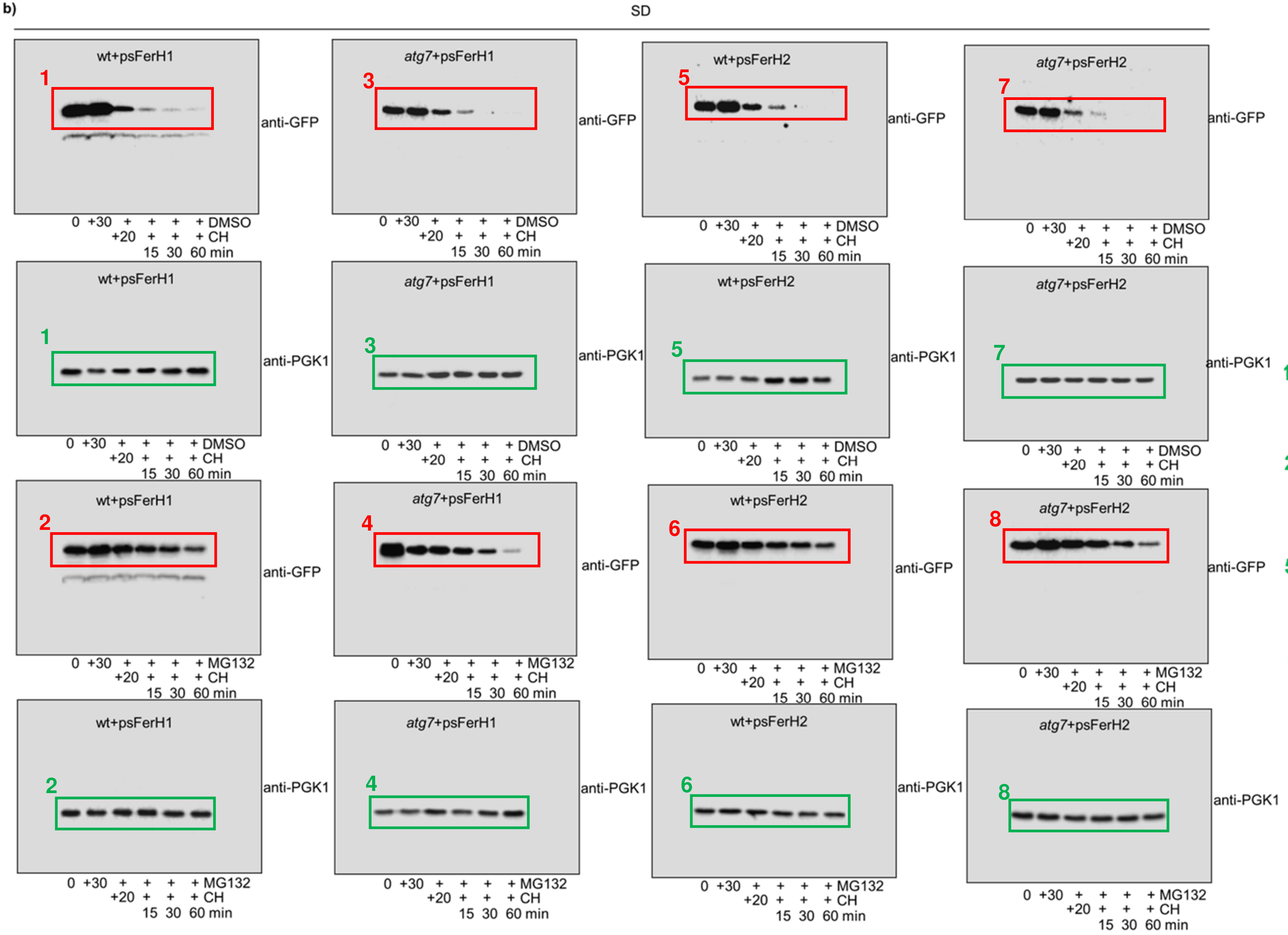

Figure 3

b)

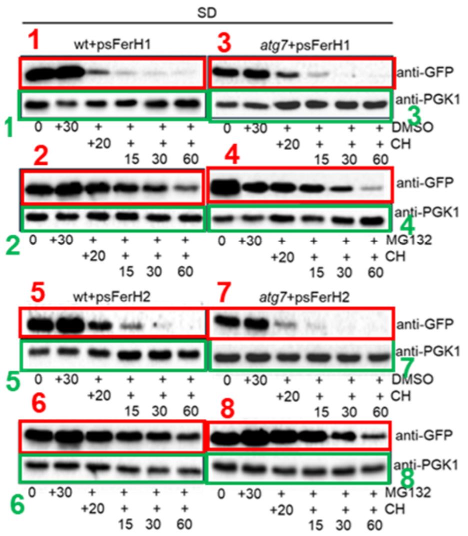

Figure 3
c)

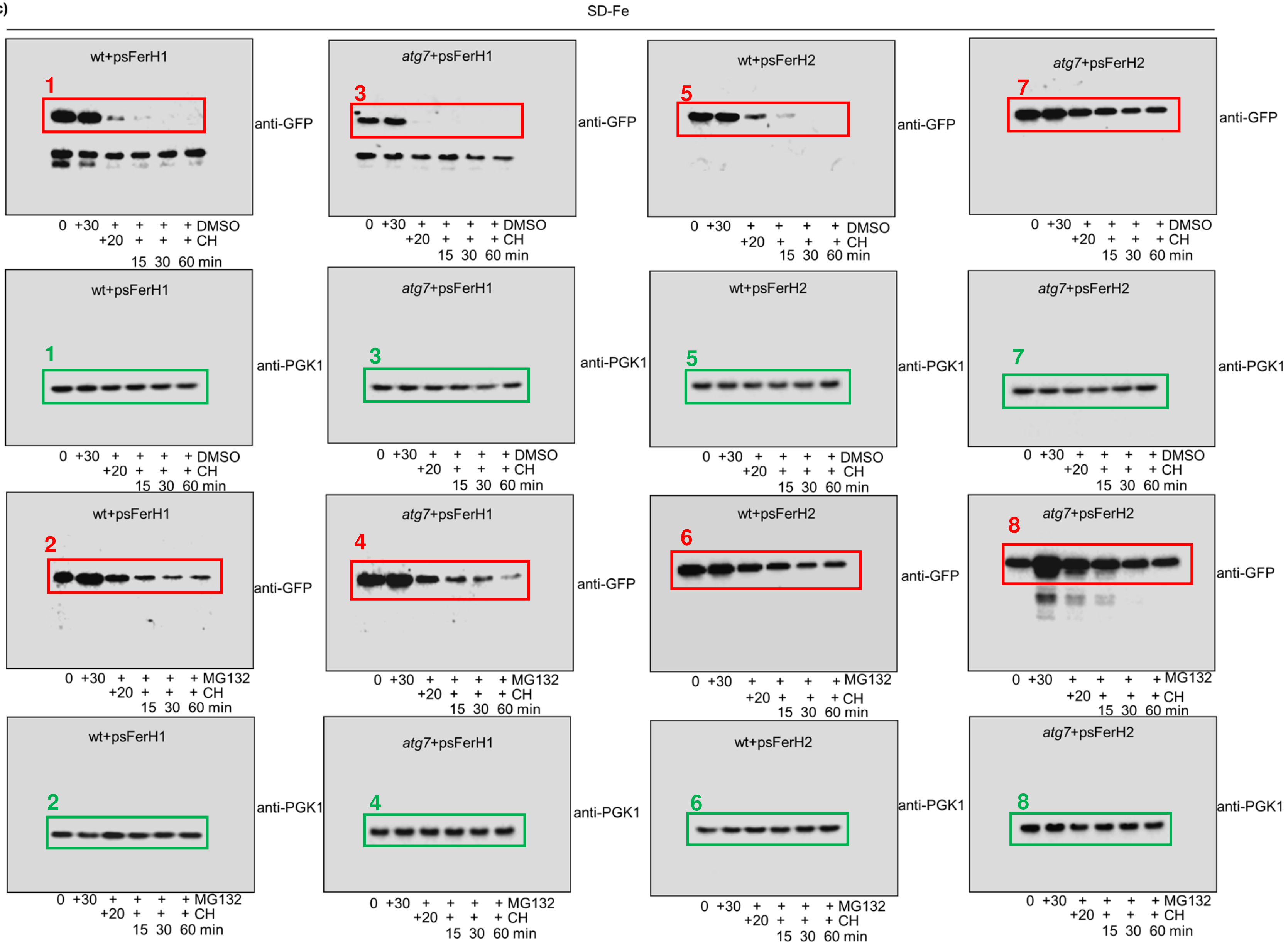

Figure 3
c)

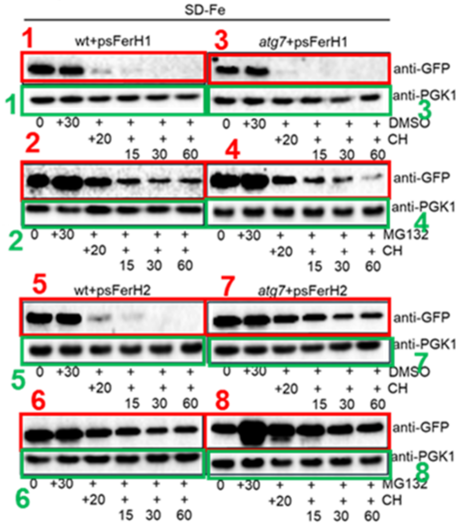

Supplementary Materials
Figure S1
a)

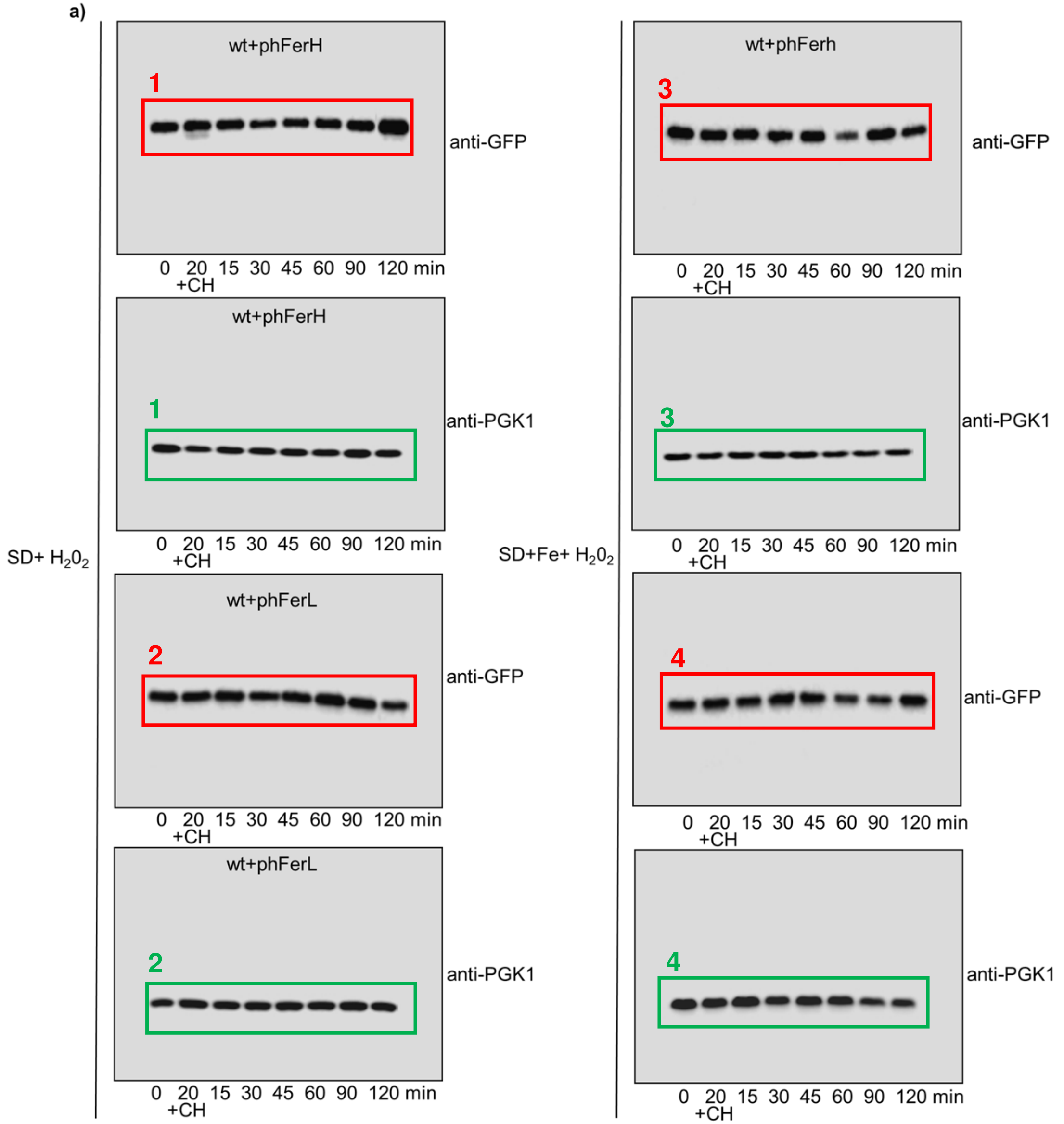

Figure S1

a)

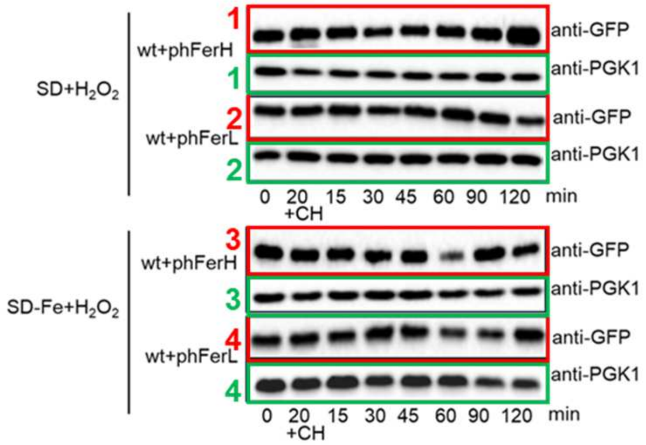

Supplementary Materials
Figure S1
b)

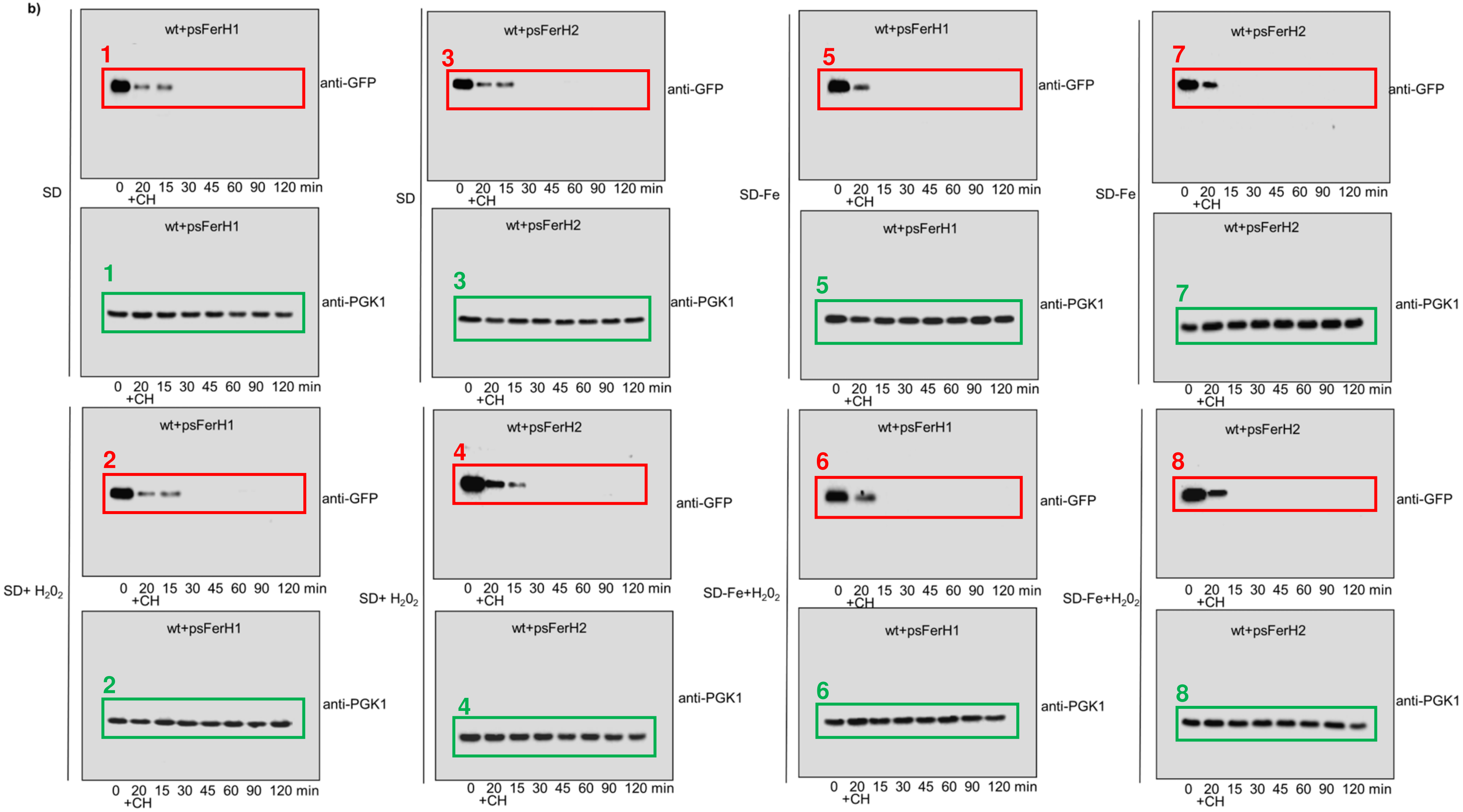

Figure S1

b)

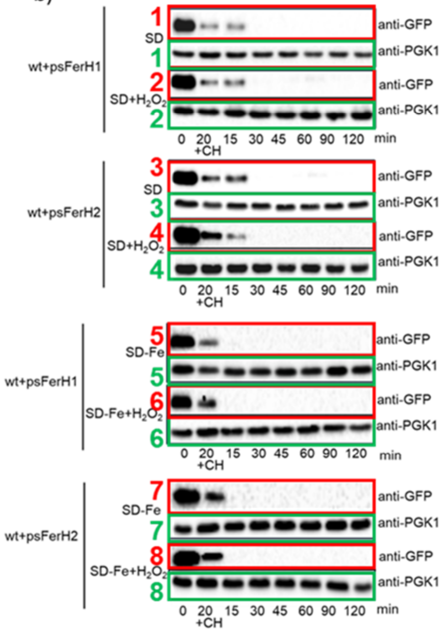

Supplement: Supplementary file 1 [file biomolecules-15-00447-s001.zip › PDF 2_Original WB Images.pdf]
