# Supplementary figures and images for: Heterologous Expression of Either Human or Soya Bean Ferritins in Budding Yeast Reveals Common Functions Protecting Against Oxidative Agents and Counteracting Double-Strand Break Accumulation
Source: Biomolecules. 2025 Mar 20;15(3):447. doi: 10.3390/biom15030447 (PMC11939973; doi:10.3390/biom15030447)

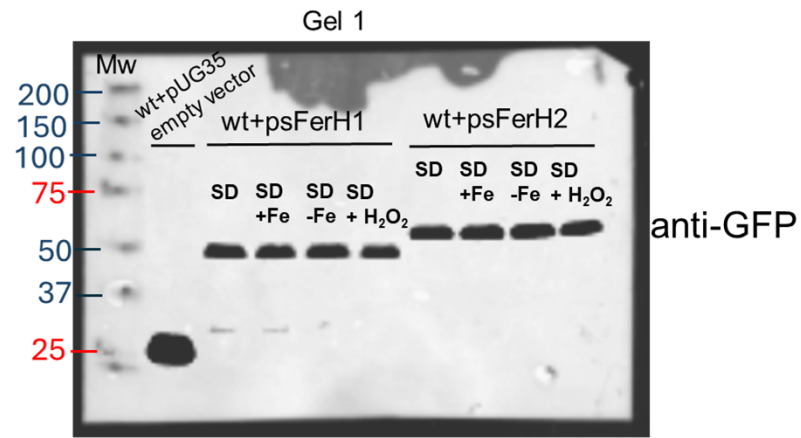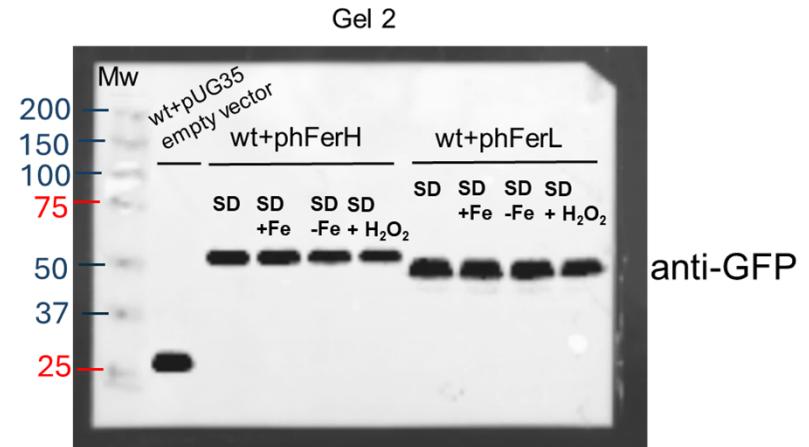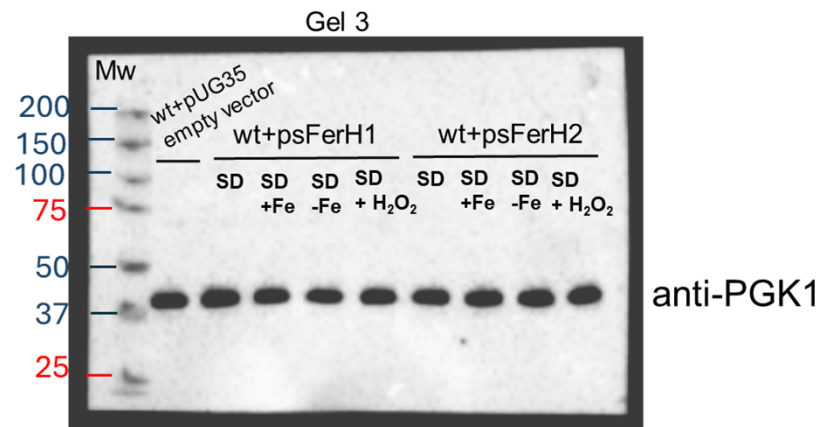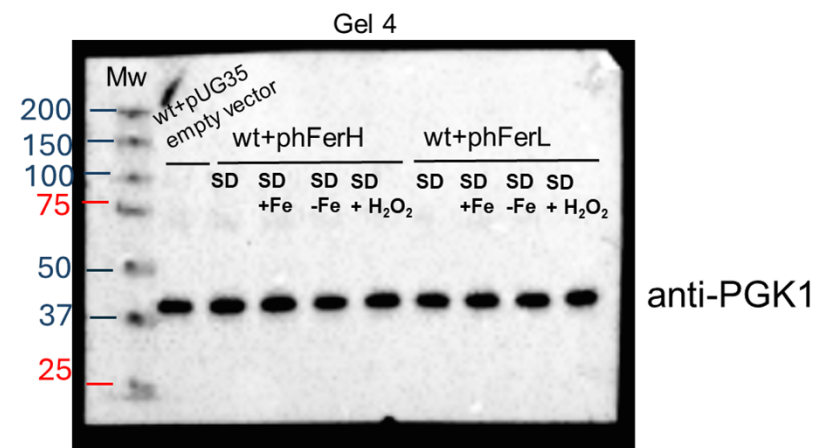

Figure 2

a)

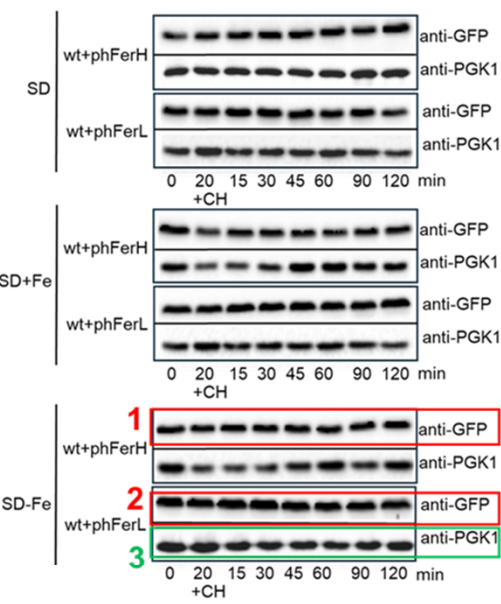

Figure 2

b)

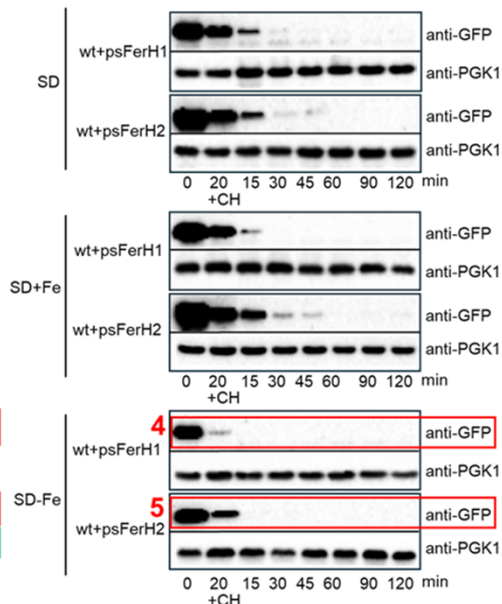

Figure 2

a)

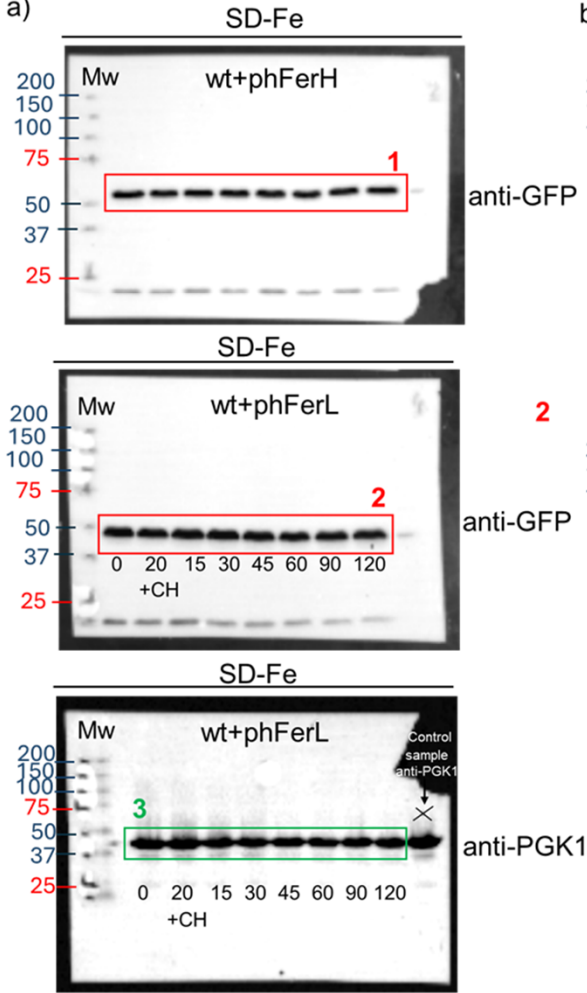

Figure 2

b)

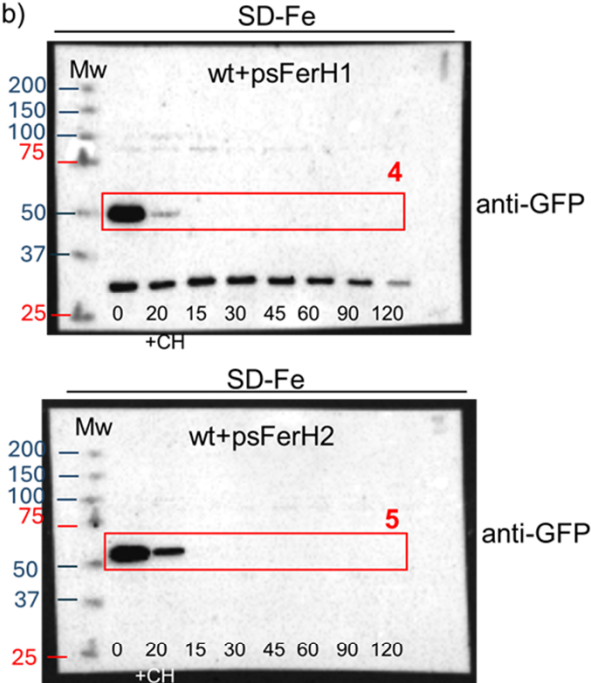

Supplement: Supplementary file 1 [file biomolecules-15-00447-s001.zip › PDF 1_Original WB images uncropped with a ladder.pdf]
